# *Acinetobacter pittii*: the emergence of a hospital-acquired pathogen analyzed from the genomic perspective

**DOI:** 10.3389/fmicb.2024.1412775

**Published:** 2024-06-26

**Authors:** Elena Bello-López, Ana Sofía Escobedo-Muñoz, Gabriela Guerrero, Ariadnna Cruz-Córdova, Elvira Garza-González, Rigoberto Hernández-Castro, Patricia Lozano Zarain, Rayo Morfín-Otero, Patricia Volkow, Juan Xicohtencatl-Cortes, Miguel A. Cevallos

**Affiliations:** ^1^Universidad Nacional Autónoma de México, Centro de Ciencias Genómicas, Programa de Genómica Evolutiva, Cuernavaca, Mexico; ^2^Universidad Nacional Autónoma de México, Centro de Ciencias Genómicas, Unidad de Análisis Bioinformáticos, Cuernavaca, Mexico; ^3^Unidad de Enfermedades Infecciosas, Laboratorio de Investigación en Bacteriología Intestinal, Hospital Infantil de México Federico Gómez, Ciudad de México, Mexico; ^4^Universidad Autónoma de Nuevo León, Facultad de Medicina/Hospital Universitario Dr. José Eleuterio González, Departamento de Bioquímica y Medicina Molecular, Monterrey, Mexico; ^5^Departamento de Ecología de Agentes Patógenos, Hospital General Dr. Manuel Gea González, Ciudad de México, Mexico; ^6^Benemérita Universidad Autónoma de Puebla, Instituto de Ciencias, Centro de Investigaciones en Ciencias Microbiológicas, Laboratorio de Microbiología Hospitalaria y de la Comunidad, Puebla, Mexico; ^7^Instituto de Patología Infecciosa y Experimental, Universidad de Guadalajara, Guadalajara, Mexico; ^8^Instituto Nacional de Cancerología, Departamento de Enfermedades Infecciosas, Ciudad de México, Mexico

**Keywords:** ESKAPE, infection, antibiotic-resistance genes, virulence, evolution

## Abstract

*Acinetobacter pittii* has increasingly been associated with several types of hospital-acquired severe infections. Genes implicated in carbapenem resistance, tigecycline resistance, or genes encoding extended spectrum cephalosporinases, such as *bla*ADC, are commonly found in isolates implicated in these infections. *A. pittii* strains that are pandrug resistant have occasionally been identified. Food for human consumption, animals and plants are environmental sources of this pathogen. An alarming situation is that *A. pitti* has been identified as responsible for outbreaks in different regions worldwide. In this study, 384 genomes of *A. pittii* were analyzed, comprising sequences from clinical and non-clinical origins from 32 countries. The objective was to investigate if clinical strains possess genetic traits facilitating hospital adaptation. Results indicate significant genomic variability in terms of size and gene content among *A. pittii* isolates. The core genome represents a small portion (25–36%) of each isolate’s genome, while genes associated with antibiotic resistance and virulence predominantly belong to the accessory genome. Notably, antibiotic resistance genes are encoded by a diverse array of plasmids. As the core genome between environmental and hospital isolates is the same, we can assume that hospital isolates acquired ARGs due to a high selective pressure in these settings. The strain’s phylogeographic distribution indicates that there is no geographical bias in the isolate distribution; isolates from different geographic regions are dispersed throughout a core genome phylogenetic tree. A single clade may include isolates from extremely distant geographical areas. Furthermore, strains isolated from the environment or animal, or plant sources frequently share the same clade as hospital isolates. Our analysis showed that the clinical isolates do not already possess specific genes, other than antibiotic-resistant genes, to thrive in the hospital setting.

## Introduction

The constant increase in bacterial strains capable of resisting a wide range of antibiotics imposes a serious problem for human health. Multidrug-resistant (MDR) bacterial pathogens are responsible for 15–50% of hospital-acquired infections worldwide (Aloke and Achilonu, 2023). Unfortunately, the problem is increasing annually. MDR bacteria are responsible for at least 700,000 deaths annually, but it is projected that 10 millions of deaths will occur by 2050 (O’Neill, 2016). With these numbers in mind, the WHO has developed a list of antibiotic-resistant pathogens to channel research leading to the discovery of new antibiotics and the development of new therapies. The most problematic bacterial pathogens within the hospital setting are known as ESKAPE microorganisms, which are known as *Enterococcus faecium*, *Staphylococcus aureus*, *Klebsiella pneumoniae*, *A. baumannii*, *Pseudomonas aeruginosa*, and *Enterobacter* species. Nonetheless, carbapenem-resistant *A. baumannii* is the WHO priority pathogen for the research and development of new antibiotics (Tacconelli et al., 2018), and it is no surprise, considering the number of outbreaks it causes annually worldwide and its mortality rate, especially in intensive care unit patients (Falagas and Rafailidis, 2007; Mohd Sazlly Lim et al., 2019; Cornejo-Juárez et al., 2020; Antimicrobial Resistance Collaborators, 2022).

Importantly, other members of the *Acinetobacter* genus could follow the same path as *A. baumannii*. Almost one-third of the species within the *Acinetobacter* genus are associated with hospital-acquired infections (Nemec, 2022). Aside from *A. baumannii, A. pittii* is becoming a dangerous emergent pathogen. This microorganism is frequently isolated from environmental sources (He and Wan, 2021; Izotova et al., 2021; Jiang et al., 2022), such as from food for human consumption offered in the markets (Carvalheira et al., 2017a,b), and a wide range of animals, including dogs, cats, birds, frogs, head lice, fishes, and humans. In these hosts, *A. pittii* is frequently found as an infection agent (Li et al., 2017; Boumbanda-Koyo et al., 2020; Wang et al., 2020; Verma et al., 2021; Cevallos et al., 2022; Leelapsawas et al., 2022; Bello-López et al., 2024).

In recent years, *A. pittii* has gradually been more frequently linked to different kinds of hospital-acquired infections, such as bloodstream infections, ventilator-associated pneumonia, and wound infections (Horrevorts et al., 1995; Brasiliense et al., 2019; Tian et al., 2022). Isolates involved in hospital-acquired infections frequently carry genes involved in carbapenem resistance, such as NDM-1, *bla*OXA58, *bla*GIM-1, *blaTEM*, or *bla*VIM-2 (Chapartegui-González et al., 2022; Wunderlich et al., 2023; Tobin et al., 2024); genes involved in tigecycline resistance (Qian et al., 2023); or genes encoding extended-spectrum cephalosporinases, such as *bla*ADC (*Acinetobacter*-derived cephalosporinases) (Hujer et al., 2005; Périchon et al., 2014). Moreover, a pandrug-resistant *A. pittii* strain has been isolated from a patient with severe pneumonia, in a Chinese hospital (Yang et al., 2021).

A sign of concern is that *A. pitti* has been identified as responsible for several outbreaks in different regions worldwide (Horrevorts et al., 1995; Idzenga et al., 2006). Furthermore, in some hospitals from Japan, France and Germany, it has been reported that *A. pittii* is the most common isolated *Acinetobacter* species (Schleicher et al., 2013; Pailhoriès et al., 2018; Kiyasu et al., 2020). All these observations drove us to study *A. pittii* closely and identify all those changes at the genomic level that are enabling it to become a worrying emerging pathogen.

To properly assess the characteristics that could render *A. pittii* as an emerging hospital-acquired pathogen, they must be contrasted with what we know about *A. baumannii. A. baumannii* is a well-established nosocomial pathogen. Most hospital-acquired infections caused by *A. baumannii* are attributed to a limited set of strains, known as international clones. These strains often exhibit resistance to a wide range of antibiotics, a feature crucial for their expansion and success in the hospital environment (Shelenkov et al., 2023). Genes involved in antibiotic resistance are linked to an ample variety of mobile genetic elements (Noel et al., 2022).

Recent research has revealed that *A. baumannii* strains isolated from non-nosocomial sources, such as grasslands, poultry, livestock, or wastewater, belong to new clones not closely associated with international clones. In other words: the international clones and the environmental isolates are grouped in different clades, strongly suggesting a differentiation between hospital-acquired isolates and those from other origins (Hamidian et al., 2022; Mateo-Estrada et al., 2022, 2023). Moreover, environmental-origin strains generally possess fewer antibiotic-resistance genes compared to international clones (Hamidian et al., 2022; Mateo-Estrada et al., 2022, 2023).

These observations also suggest that strains belonging to the international clones already possess specializations that enable them to survive and thrive in the hospital environment. This also implies that members of the international clones have little contact with environmental strains and prefer to exchange genetic material among themselves. A clear sign of their adaptation to the hospital setting is that members of the international clones easily spread between hospitals and even continents via infected patients; once a clone invades a hospital, eradicating it becomes extremely difficult (Peleg et al., 2008; Zarrilli et al., 2009; Birgand et al., 2014; Cornejo-Juárez et al., 2020; Higgins et al., 2021). Another important feature for survival in the hospital setting is that *A. baumannii* isolates can resist desiccation and frequently form biofilms (Lucidi et al., 2024).

With this in mind, we analyzed a total of 384 high quality genomic sequences. 352 downloaded from NCBI, and 32 *A. pittii* strains sequenced by us. 29 of them are nosocomial strains isolated from 7 hospitals, six Mexicans and one Honduran hospital. We also sequenced three strains isolated from animals. The collection included *A. pittii* isolates samples from 32 countries. Here, we show that the *A. pittii* genome varies widely in terms of genome size and gene content. The core genome is a small fraction (25 to 36%) of the genome of each *A. pittii* isolate. Importantly, most of the genes involved in antibiotic resistance or virulence are part of the accessory genome. The majority of the genes involved in antibiotic resistance are encoded by a vast range of plasmids. The strains’ phylogeographic distribution indicates no geographical bias in the isolate distribution; isolates from different geographic regions—even continents—are dispersed throughout a core genome phylogenetic tree. A single clade may include isolates from extremely distant geographical areas. Furthermore, strains isolated from the environment or animal/plant sources frequently share the same clade as hospital isolates. Our analysis showed that the clinical isolates do not already possess specific genes, other than antibiotic-resistant genes, to thrive in the hospital setting.

## Results

### Phylogeographic distribution of *A. pittii* isolates

To analyze the geographical distribution from a phyletic perspective, we constructed a maximum likelihood phylogenetic tree with 352 high-quality *A. pittii* genome assemblies downloaded from NCBI plus 29 genome sequences from Mexican and Honduran hospital-acquired isolates and three genome sequences obtained from animals (Table 1, Supplementary Table S1). We used an *A. baumannii* genome as the outgroup sequence. Importantly, these sequences came from 32 countries from very different regions; nevertheless, the collection had a high sample bias considering that most of the genomes came from three countries: China, Germany, and the United States. Moreover, most of the genome sequences were obtained from clinical isolates (82%). The collection also contained strains obtained from non-clinical samples: soil/water (24 strains), plants (2 strains), animals (17 strains), fomites (4 strains) and from the International Space Station (21 strains).

**Table 1:** Strains sequenced in this work.

BioSample	Accession_num. NCBI	Strain	Collected_by	Source	Country: province, city
SAMN38315714	GCF_033952765.1	34H	Hospital Infantil Federico Gomez	Nosocomial	Mexico: Mexico city
SAMN38315715	GCF_033952265.1	539 U	Hospital Infantil Federico Gomez	Nosocomial	Mexico: Mexico city
**SAMN38315702**	**GCF_034070325.1**	**564 U**	**Hospital Infantil Federico Gomez**	Nosocomial	**Mexico: Mexico city**
**SAMN38315703**	**GCF_034067285.1**	**AbaK**	**Hospital General Doctor Manuel Gea Gonzalez**	Nosocomial	**Mexico: Mexico city**
**SAMN38315704**	**GCF_034073245.1**	**AE13**	**Hospital Infantil Federico Gomez**	Nosocomial	**Mexico: Mexico city**
**SAMN38315705**	**GCF_034072545.1**	**AN37**	**Hospital paral niño Poblano**	Nosocomial	**Mexico:Puebla, Puebla**
SAMN38315716	GCF_033952175.1	AN38	Hospital para niño Poblano	Nosocomial	Mexico:Puebla, Puebla
SAMN38315717	GCF_033952235.1	AN42	Hospital para niño Poblano	Nosocomial	Mexico:Puebla, Puebla
SAMN38315718	GCF_033952745.1	AN51	Hospital para niño Poblano	Nosocomial	Mexico:Puebla, Puebla
**SAMN38315706**	**GCF_034071365.1**	**HCG138**	**Hospital Civil de Guadalajara**	Nosocomial	**Mexico: Jalisco, Guadalajara**
**SAMN38315730**	**GCF_034063485.1**	**HCG18**	**Hospital Civil de Guadalajara**	Nosocomial	**Mexico: Jalisco, Guadalajara**
**SAMN38315707**	**GCF_034071845.1**	**HCG62**	**Hospital Civil de Guadalajara**	Nosocomial	**Mexico: Jalisco, Guadalajara**
**SAMN38315709**	**GCF_034068265.1**	**HUM1**	**Hospital Universitario Dr. Jose Eleuterio Gonzalez**	Nosocomial	**Mexico: NuevoLeon, Monterey**
SAMN38315719	GCF_033952725.1	HUM10	Hospital Universitario Dr. Jose Eleuterio Gonzalez	Nosocomial	Mexico: NuevoLeon, Monterey
SAMN38315720	GCF_033952685.1	HUM11	Hospital Universitario Dr. Jose Eleuterio Gonzalez	Nosocomial	Mexico: NuevoLeon, Monterey
SAMN38315721	GCF_033952225.1	HUM12	Hospital Universitario Dr. Jose Eleuterio Gonzalez	Nosocomial	Mexico: NuevoLeon, Monterey
SAMN38315722	GCF_033952285.1	HUM13	Hospital Universitario Dr. Jose Eleuterio Gonzalez	Nosocomial	Mexico: NuevoLeon, Monterey
**SAMN38315708**	**GCF_034069865.1**	**HUM14**	**Hospital Universitario Dr. Jose Eleuterio Gonzalez**	Nosocomial	**Mexico: NuevoLeon, Monterey**
SAMN38315723	GCF_033952165.1	HUM2	Hospital Universitario Dr. Jose Eleuterio Gonzalez	Nosocomial	Mexico: NuevoLeon, Monterey
**SAMN38315710**	**GCF_034066905.1**	**HUM3**	**Hospital Universitario Dr. Jose Eleuterio Gonzalez**	Nosocomial	**Mexico: NuevoLeon, Monterey**
SAMN38315724	GCF_033952185.1	HUM4	Hospital Universitario Dr. Jose Eleuterio Gonzalez	Nosocomial	Mexico: NuevoLeon, Monterey
SAMN38315725	GCF_034044015.1	HUM5	Hospital Universitario Dr. Jose Eleuterio Gonzalez	Nosocomial	Mexico: NuevoLeon, Monterey
SAMN38315726	GCF_033952705.1	HUM6	Hospital Universitario Dr. Jose Eleuterio Gonzalez	Nosocomial	Mexico: NuevoLeon, Monterey
SAMN38315727	GCF_033952625.1	HUM7	Hospital Universitario Dr. Jose Eleuterio Gonzalez	Nosocomial	Mexico: NuevoLeon, Monterey
SAMN38315728	GCF_033952605.1	HUM8	Hospital Universitario Dr. Jose Eleuterio Gonzalez	Nosocomial	Mexico: NuevoLeon, Monterey
SAMN38315729	GCF_033952565.1	INC1094	Instituto Nacional de Cancerologia	Nosocomial	Mexico: Mexico city
**SAMN38315711**	**GCF_034066105.1**	**MCR16048**	**Hospital Catarino Rivas**	Nosocomial	**Honduras: Cortes, San Pedro Sula**
**SAMN38315712**	**GCF_034068865.1**	**MCR53**	**Hospital Catarino Rivas**	Nosocomial	**Honduras: Cortes, San Pedro Sula**
**SAMN38315713**	**GCF_034064985.1**	**MCR8900**	**Hospital Catarino Rivas**	Nosocomial	**Honduras: Cortes, San Pedro Sula**
SAMN33416577	GCF_028890465.1	978-A_19	Dr. Rigoberto Hernández Castro (HGDMGG)	Environmental (red-lored parrot)	Mexico: Mexico city
SAMN28658287	GCF_023669665.1	A47H	Dr. Eria Rebollar (UNAM)	Environmental (frog)	Panama: Gamboa
**SAMN28658286**	**GCF_023669645.1**	**A45P**	**Dr. Eria Rebollar (UNAM)**	**Environmental (frog)**	**Panama: Gamboa**

GenBank, Biosample, and accession numbers of the strains sequenced in this work. Strains marked in bold letters represent closed genomes. HGDMGG, Hospital General Doctor Manuel Gea Gonzalez. UNAM, Universidad Nacional Autónoma de México.

The genome size of *A. pittii* varied greatly: The smallest genome was 3.57 Mb, and the largest genome was 4.5 Mb. In concordance, the number of proteins encoded in the *A. pittii* genomes studied here also showed a high degree of variation, ranging from 3,149 to 4,198. The *A. pittii,* genes which are from strict core genome*s* consist of 1,102 families of orthologous proteins, and they are involved mainly in (A) Translation, ribosomal structure, and biogenesis. (B) Amino acid transport and metabolism. (C) Energy production and conversion. (D) Coenzyme transport and metabolism, and (E) Cell wall, membrane a and envelope biogenesis.

**Figure 1:**
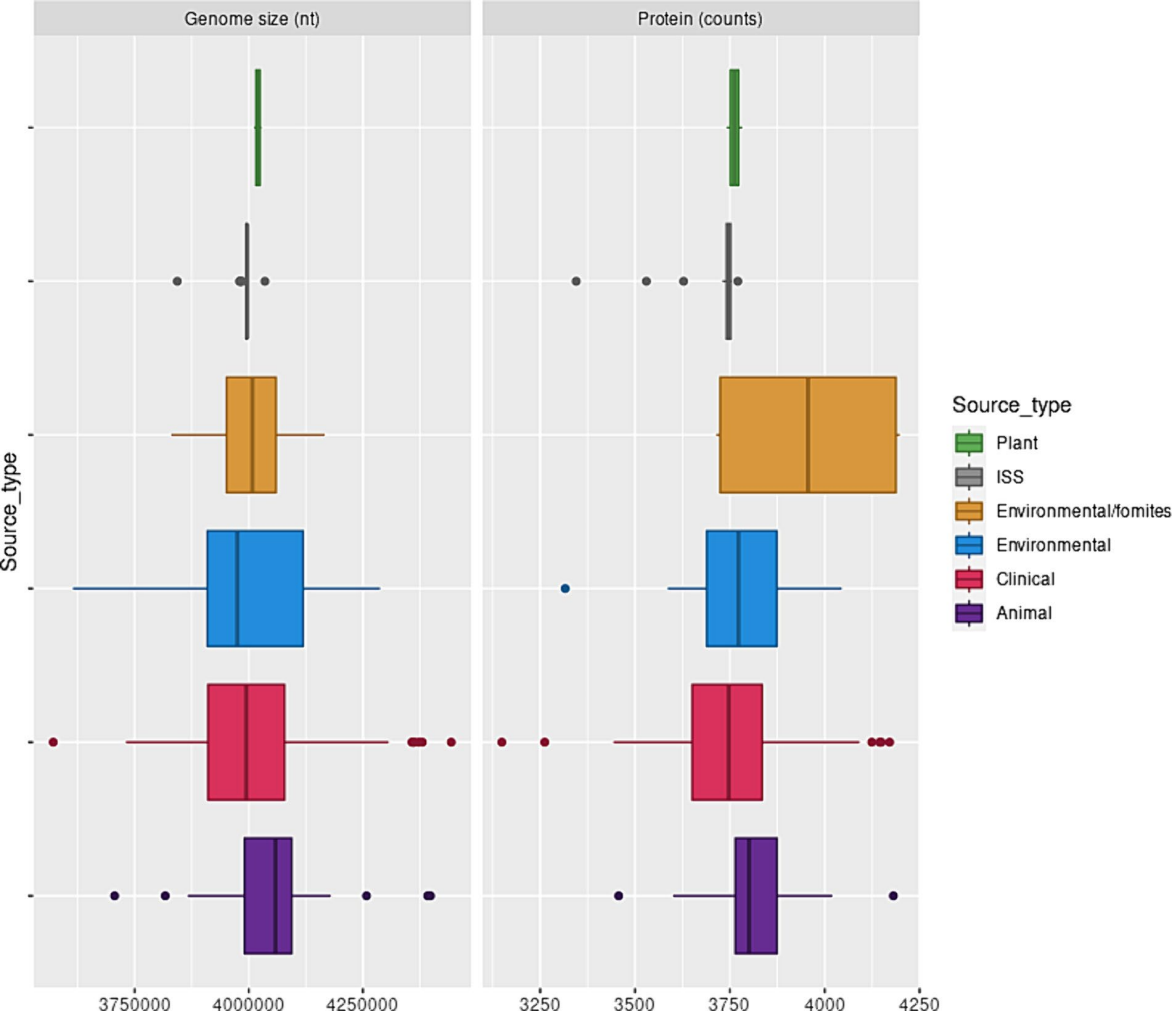
*A. pittii* genome sizes and protein counts. Box and whisker plot showing the distribution of *A. pittii* genome sizes and protein counts considering isolation source. Clinical: Median, 3994436.0; Mean, 4001563.8; sd, 134450.5. Animal: Median, 4058504.5; Mean, 4062697.7; sd, 175490.6. Environmental: Median, 3974814.5; Mean, 3991148.0; sd, 158,914. Environmental/Fomites: Median, 4007841.0; Mean, 4002936.0; sd, 138086.1. ISS: Median, 3995985.0; Mean, 3988077.52; sd, 34882.89. Plant: Median, 4,019,960; Mean, 4,019,960; sd, 119.89.

This observation also implied that the strict core genome of each isolate represented between 25 and 36% of the genome. The genome size and the number of proteins they encoded did not depend on the isolation source (Figure 1). The pangenome of the *A. pittii* isolates analyzed here consisted of 32,019 proteins, indicating its ecological versatility. To evaluate the diversity of *A. pittii*, we analyzed SNVs of 279 core genes that did not exhibit recombination signals after eliminating paralogs. This analysis revealed that *A. pittii* was highly diverse; the average pairwise distance was 1,698 SNVs, and the largest difference between the two strains was 4,214 SNVs (Supplementary Figure S1).

**Figure 2:**
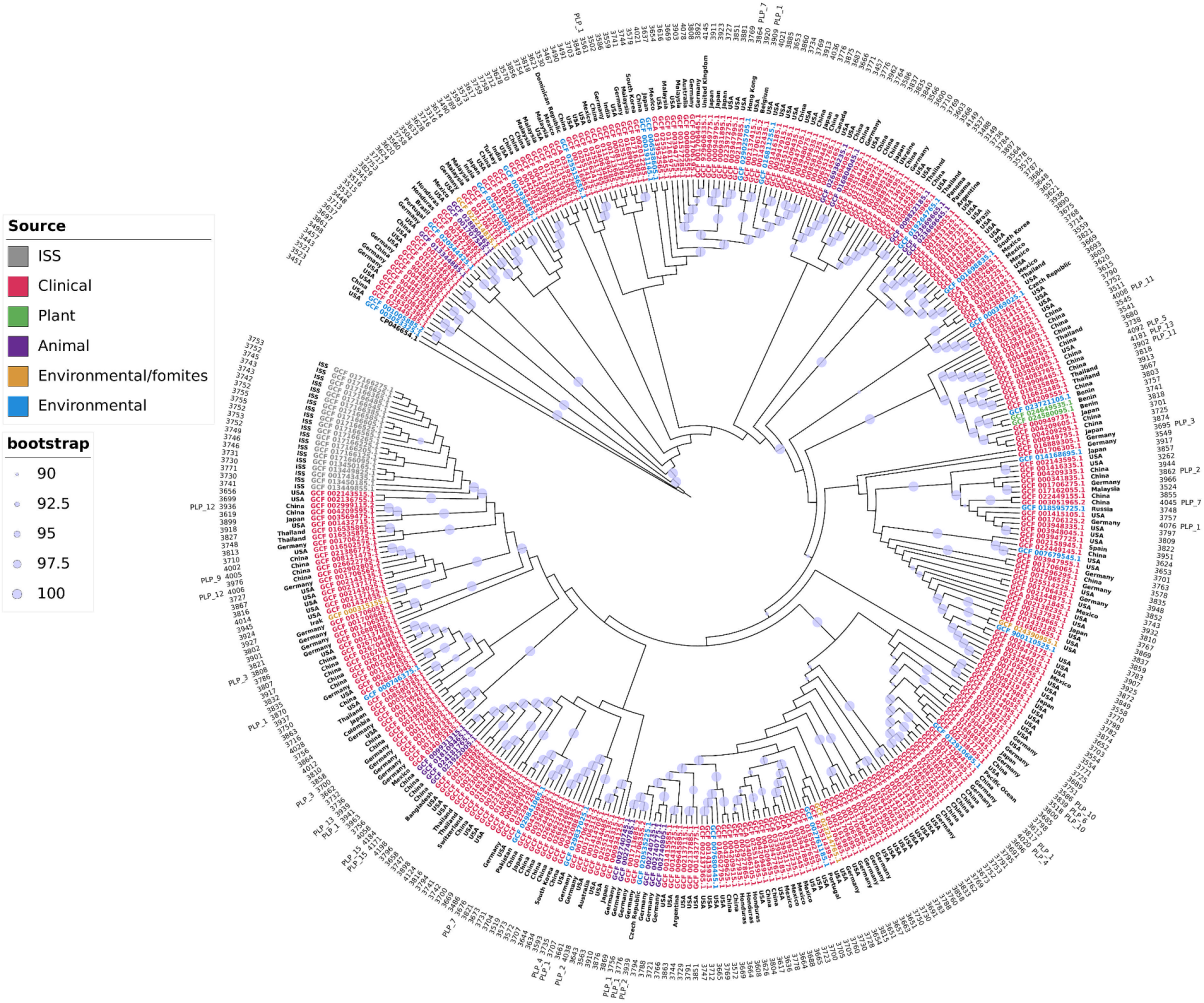
*A. pittii* core genome phylogenetic tree. Maximum likelihood phylogenetic tree of *A. pittii* constructed with strict core genome genes of isolates obtained from clinical, environmental, environmental fomites, animal, plant, and International Space Station (ISS) sources. *A. baumannii* (CP046654.1) was used as outgroup. External circle indicates strains with plasmids belonging to a plasmid lineage (PLP). Numbers in the next inner circle shows the number of proteins of each isolate. The next circle shows the country of origin of each isolate. The Maximum likelihood phylogenetic tree considering distances is presented in Supplementary Figure S2.

Two important observations could be drawn from the tree topology presented in Figure 2 and Supplementary Figure S2. First, the distribution of the isolates did not have a geographical bias; isolates from distinct geographical regions, even continents, were interspersed along the tree. The same clade could include isolates from distant geographical regions. For example, strains UKK-0145 (Turkey), ABC (India), ABBL019 (USA), WCHAP00001, WCHAP00003, and WCHAP00021 (China) were grouped in the same clade. The only exception we found was that the strains isolated from the International Space Station comprised a single clade, indicating recent clonal expansion. These observations indicated that the *A. pittii* isolates were subject to frequent intercontinental introductions. In other words, *A. pittii* did not seem to have obvious migration barriers. The second noteworthy observation was that we did not find specific clones associated with specific habitats. Moreover, clinical isolates frequently shared the same clade as strains obtained from environmental and/or animal or plant sources. One possible interpretation of this observation was that all strains of *A. pittii* could be pathogenic if they encountered the right host, usually immunocompromised, and the route to invade it.

The phylogenetic tree presented here was constructed from genes that constituted the core genome of this species but as stated above, represented a small percentage of the genes *A. pittii* genome. To determine whether the accessory genome, the largest fraction of the genome of this bacterium, could be used to group strains by geography or by isolation source, we constructed a phylogenetic tree based on a matrix of the presence and absence of genes. This phylogenetic tree (Supplementary Figure S3) shows that excluding the genomes obtained from the International Space Station, the strains were not associated with a location or an isolation source.

### Antibiotic resistance genes

To identify the genes related to acquired antibiotic present in *A. pittii* genomes, we consulted the Comprehensive Antibiotic Resistance Database (CARD) (Alcock et al., 2020; Florensa et al., 2022). The result of this analysis is presented in Supplementary Figure S4. However, we did the same exercise to determine in which fraction of the genome (core or accessory) the genes were located. We found only four genes in the core genome with matches in this database: The small multidrug resistance (SMR) antibiotic efflux pump AbeS, the resistance-nodulation-cell division (RND) antibiotic efflux pump AdeF, the intrinsic peptide antibiotic-resistant Lps, and the *bla*ADC *beta*-lactamase. Nevertheless, it is important to consider that all strains possess a *bla*OXA gene, but they are different enough to belong to distinct families of orthologous proteins. The most common *bla*OXA genes belong to the *bla*OXA-213 family (*bla*OXA500). On the other hand, we obtained 71 matches in the CARD using the *A. pittii* accessory genome as a query (Supplementary Table S2). As expected, many of these genes were encoded in plasmids (Table 2).

**Table 2:** *A. pittii* plasmids.

	Accession_number	Strain	Plasmid_name	Plasmid-size	Rep_protein	Accessions_CDD	Protein_ID	Lineage
1	**NZ_AGFH01000030.1**	**D499**	**pAB_D499**	**47,101**	**NI**			**PLP_1**
2	**NZ_CM001802.1**	**XM1570**	**pMX1**	**47,274**	**NI**			**PLP_1**
3	**NZ_MDHV02000003.1**	**UKK-0265**	**pGIM1_UKK-0265**	**39,562**	**NI**			**PLP_1**
4	**NZ_MDHX02000003.1**	**UKK-0432**	**pNDM1_UKK-0432**	**48,580**	**NI**			**PLP_1**
5	**NZ_MDIR02000003.1**	**UKK-0553**	**pGIM1_UKK-0553**	**43,536**	**NI**			**PLP_1**
6	**NZ_MDIS02000003.1**	**UKK-0554**	**pGIM1_UKK-0554**	**42,583**	**NI**			**PLP_1**
7	**NZ_MDIB02000003.1**	**UKK-0538**	**pNDM1_UKK-0538**	**47,278**	**NI**			**PLP_1**
8	**NZ_MDIU02000005.1**	**UKK-0556**	**pGIM1_UKK-0556**	**43,772**	**NI**			**PLP_1**
9	**NZ_MDIC02000004.1**	**UKK-0539**	**pNDM1_UKK-0539**	**49,842**	**NI**			**PLP_1**
10	**NZ_MDIK02000003.1**	**UKK-0547**	**pVIM2_UKK-0547**	**46,530**	**NI**			**PLP_1**
11	**NZ_MDIM02000003.1**	**UKK-0548**	**pVIM2_UKK-0548**	**45,234**	**NI**			**PLP_1**
12	**NZ_MDID02000002.1**	**UKK-0540**	**pNDM1_UKK-0540**	**46,164**	**NI**			**PLP_1**
13	**NZ_MDIT02000009.1**	**UKK-0555**	**pGIM1_UKK-0555**	**42,583**	**NI**			**PLP_1**
14	NZ_CM001803.1	XM1570	pMX2	93,891	Rep_3-DUF5710	pfam01051,cl44662	WP_000064928.1	PLP_2
15	NZ_CP026087.2	WCHAP005069	p1_005069	91,563	Rep_3-DUF5710	pfam01051,cl44662	WP_000064928.1	PLP_2
16	NZ_CP027247.2	WCHAP100004	p1_100004	66,765	Rep_3-DUF5710	pfam01051, cl44662	WP_000064928.1	PLP_2
17	NZ_CP027251.3	WCHAP100020	p1_100020	77,340	Rep_3-DUF5710	pfam01051, cl44662	WP_000064928.1	PLP_2
18	NZ_CP042366.1	C54	pC54_002	76,008	Rep_3-DUF5710	pfam01051, cl44662	WP_000064928.1	PLP_2
19	NZ_CP043054.1	AP43	pAP43-2	92,276	Rep_3-DUF5710	pfam01051, cl44662	WP_000064928.1	PLP_2
20	NZ_CP069506.1	FDAARGOS_1215	unnamed2	94,379	Rep_3-DUF5710	pfam01051, cl44662	WP_000064928.1	PLP_2
21	NZ_CP069540.1	FDAARGOS_1214	unnamed3	94,387	Rep_3-DUF5710	pfam01051, cl44662	WP_000064928.1	PLP_2
22	NZ_CP077304.1	FDAARGOS_1396	unnamed1	94,386	Rep_3-DUF5710	pfam01051,cl44662	WP_000064928.1	PLP_2
23	NZ_CP139277.1	AN37	pApiAN37f	85,134	Rep_3-DUF5711	pfam01051,cl44663	WP_000064928.1	PLP_2
24	NZ_CP084922.1	CEP14	pCEP14_01	95,483	Rep_3-DUF5710	pfam01051, cl44662	WP_000064928.1	PLP_2
25	NZ_CP027252.3	WCHAP100020	p2_100020	9,132	RepM_Acin	cl45741	WP_005804946.1	PLP_3
26	NZ_CP069497.1	FDAARGOS_1217	unnamed1	9,060	RepM_Acin	cI45741	WP_005804946.1	PLP_3
27	NZ_CP069507.1	FDAARGOS_1215	unnamed3	9,084	RepM_Acin	cl45741	WP_005804946.1	PLP_3
28	NZ_CP069538.1	FDAARGOS_1214	unnamed1	9,132	RepM_Acin	cl45741	WP_005804946.1	PLP_3
29	NZ_CP077306.1	FDAARGOS_1396	unnamed3	9,038	NI			PLP_3
30	NZ_CP118935.1	ML4	pML4-2	9,131	RepM_Acin	cI45741	WP_005804946.1	PLP_3
31	NZ_MDIB02000004.1	UKK-0538	p4_UKK-0538	8,804	NI			PLP_4
32	NZ_MDIK02000005.1	**UKK-0547**	**p5_UKK-0547**	**8,804**	**NI**			**PLP_4**
33	NZ_MDIM02000007.1	**UKK-0548**	**p7_UKK-0548**	**8,804**	**NI**			**PLP_4**
34	NZ_CP029611.1	**ST220**	**unnamed**	**84,302**	**RepM_Acin**	**cl45741**	**WP_002046604.1**	**PLP_5**
35	NZ_CP095408.1	**TCM**	**pTCM-1**	**84,108**	**RepM_Acin**	**cI45741**	**WP_002046604.1**	**PLP_5**
36	NZ_CP123766.1	**AP8900**	**pAP8900-1**	**86,394**	**RepM_Acin**	**cI45741**	**WP_002046604.1**	**PLP_5**
37	NZ_CP069505.1	FDAARGOS_1215	unnamed1	11,158	RepM_Acin	cl45741	WP_005065049.1	PLP_6
38	NZ_CP077240.1	FDAARGOS_1399	unnamed2	9,581	RepM_Acin	cl45741	WP_005065049.1	PLP_6
39	NZ_CP139289.1	564 U	pApi564 Ua	11,158	RepM_Acin	cl45741	WP_005065049.1	PLP_6
40	NZ_CP139273.1	HCG62	pApiHCG62b	11,158	RepM_Acin	cl45741	WP_005065049.1	PLP_6
41	NZ_CP139256.1	MCR16048	pApiMCR16048b	11,158	RepM_Acin	cl45741	WP_005065049.1	PLP_6
42	NZ_AP024801.1	OCU_Ac17	pOCUAc17-3	11,158	RepM_Acin	cI45741	WP_005065049.1	PLP_6
43	NZ_CP028569.1	WCHAP005046	p1_005046	121,612	RepM_Acin	cl45741	WP_000818856.1	PLP_7
44	NZ_CP077239.1	FDAARGOS_1399	unnamed1	108,027	RepM_Acin	cl45741	WP_000818856.1	PLP_7
45	NZ_CP118934.1	ML4	pML4-1	99,281	RepM_Acin	cl45741	WP_000818856.1	PLP_7
46	NZ_CP095409.1	TCM	pTCM-2	11,346	RepM_Acin	cI45741	WP_005133531.1	PLP_8
47	NZ_CP123769.1	AP8900	pAP8900-4	11,346	RepM_Acin	cI45741	WP_005133531.1	PLP_8
48	NZ_CP026088.1	WCHAP005069	p2_005069	9,203	RepM_Acin	cl45741	WP_000845851.1	PLP_9
49	NZ_CP043055.1	AP43	pAP43-3	9,203	RepM_Acin	cl45741	WP_000845851.1	PLP_9
50	NZ_CP069508.1	FDAARGOS_1215	unnamed4	11,469	RepM_Acin	cI45741	WP_005237331.1	PLP_10
51	NZ_CP077305.1	FDAARGOS_1396	unnamed2	11,469	RepM_Acin	cl45741	WP_005237331.1	PLP_10
52	NZ_CP087718.1	**AP2044**	**pAP2044-2**	**43,577**	**RepM_Acin**	**cl45741**	**WP_004843977.1**	**PLP_11**
					**CyRepA1**	**cl26703, cl28734**	**WP_069120316**	
53	NZ_CP123767.1	**AP8900**	**pAP8900-2**	**43,600**	**RepM_Acin**	**cI45741**	**WP_004843977.1**	**PLP_11**
54	NZ_CP026086.2	**WCHAP005069**	**pOXA58_005069**	**112,436**	**RepM_Acin**	**cl45741**	**WP_000845851.1**	**PLP_12**
					**RepM_Acin**	**cl45741**	**WP_002046604.1**	
55	NZ_CP027249.2	**WCHAP100004**	**pOXA58_100004**	**105,591**	**RepM_Acin**	**cl45741**	**WP_002046604.1**	**PLP_12**
56	NZ_MDIK02000007.1	**UKK-0547**	**p7_UKK-0547**	**6,078**	**NI**			**PLP_13**
57	NZ_CP095411.1	**TCM**	**pTCM-4**	**6,078**	**NI**			**PLP_13**
58	NZ_CP027660.1	Ap-W20	pApW20-2	147,574	RepM_Acin	cI45741	WP_002046604.1	PLP_14
59	NZ_CP029007.1	AbW39	pAbW39-2	149,504	RepM_Acin	cI45741	WP_005065049.1	PLP_14
					RepM_Acin	cI45741	WP_002046604.1	
60	NZ_CP027659.1	**Ap-W20**	**pApW20-1**	**237,519**	**NI**			**PLP_15**
61	NZ_CP029006.1	**AbW39**	**pAbW39-1**	**262,817**	**NI**			**PLP_15**
62	NZ_CP021429.1	HUMV-6483	p11	112,604	RepM_Acin	cl45741	WP_002046604.1	PLP_16
63	NZ_CP139267.1	HUM14	pApiHUM14c	115,839	RepM_Acin	cl45741	WP_002046604.1	PLP_16
64	NZ_AP021938.1	WP2-W18-ESBL-11	pWP2-W18-ESBL-11_2	10,416	RepM_Acin	cl45741	WP_182918118.1	PLP_17
65	NZ_CP139260.1	HUM3	pApiHUM3b	10,476	RepM_Acin	cl45741	WP_005065049.1	PLP_17
66	NZ_AP021937.1	WP2-W18-ESBL-11	pWP2-W18-ESBL-11_1	87,428	RepM_Acin	cI45741	WP_002046604.1	ORPHAN
67	NZ_AP021939.1	WP2-W18-ESBL-11	pWP2-W18-ESBL-11_3	2,726	Rep_1	cl2412	WP_001180320.1	ORPHAN
68	NZ_AP024799.1	OCU_Ac17	pOCUAc17-1	69,156	Rep_3-DUF5710	pfam01051, cl44662	WP_004795963.1	ORPHAN
69	NZ_AP024800.1	OCU_Ac17	pOCUAc17-2	15,601	RepM_Acin	cl45741	WP_004763412.1	ORPHAN
70	NZ_CP014478.1	**AP_882**	**pNDM-AP_882**	**146,597**	**NI**			**ORPHAN**
71	NZ_CP014479.1	**AP_882**	**pOXA58-AP_882**	**36,862**	**RepM_Acin**	**cl45741**	**WP_005804946.1**	**ORPHAN**
72	NZ_CP015146.1	**IEC338SC**	**pIEC338SCOX**	**10,498**	**RepM_Acin**	**cl45741**	**WP_012780181.1**	**ORPHAN**
73	NZ_CP015147.1	IEC338SC	pIEC338SC2	5,562	RepM_Acin	cl45741	WP_063099738.1	ORPHAN
74	NZ_CP015148.1	IEC338SC	pIEC338SC3	5,813	RepM_Acin	cl45741	WP_063099746.1	ORPHAN
75	NZ_CP017939.1	YMC2010/8/T346	unnamed1	28,641	II	cl31812	WP_078220828.1	ORPHAN
76	NZ_CP018910.1	**XJ88**	**unnamed1**	**9,501**	**NI**			**ORPHAN**
77	NZ_CP027248.1	WCHAP100004	p2_100004	18,485	RepM_Acin	cl45741	WP_004698334.1	ORPHAN
78	**NZ_CP027253.1**	**WCHAP100020**	**pOXA58_100020**	**24,018**	**RepM_Acin**	**cl45741**	**WP_002046604.1**	**ORPHAN**
79	NZ_CP027661.1	Ap-W20	pApW20-3	8,123	RepM_Acin	cI45741	WP_252618463.1	ORPHAN
80	NZ_CP027662.1	Ap-W20	pAP2044-4	7,680	RepM_Acin	cI45741	WP_005065049.1	ORPHAN
81	NZ_CP028570.1	WCHAP005046	p2_005046	10,472	RepM_Acin	cl45741	WP_005804946.1	ORPHAN
82	NZ_CP028571.1	WCHAP005046	p3_005046	4,771	Replicase-PriCT-HTH-23	cl03886, cl07362, pfam13384	WP_057082420.1	ORPHAN
83	NZ_CP028572.2	WCHAP005046	p4_005046	2,295	Rep_1	cl2412	WP_032071712.1	ORPHAN
84	**NZ_CP028573.2**	**WCHAP005046**	**pOXA58_005046**	**61,751**	**RepM_Acin**	**cl45741**	**WP_002046604.1**	**ORPHAN**
85	**NZ_CP040912.1**	**AB17H194**	**pAB17H194–1**	**88,002**	**Rep_3-DUF5710**	**pfam01051, cl44662**	**WP_150378260.1**	**ORPHAN**
86	NZ_CP040913.1	AB17H194	pAB17H194–2	76,962	RepM_Acin	cl45741	WP_002046604.1	ORPHAN
87	**NZ_CP042365.1**	**C54**	**pC54_001**	**256,887**	**NI**			**ORPHAN**
88	NZ_CP042367.1	C54	pC54_003	25,906	RepM_Acin	cl45741	WP_114225253.1	ORPHAN
89	NZ_CP042368.1	C54	pC54_004	6,575	Rep_3	pfam01051	WP_001031297.1	ORPHAN
90	NZ_CP042369.1	C54	pC54_005	4,478	NI			ORPHAN
91	**NZ_CP043053.1**	**AP43**	**pAP43-OXA58-NDM1**	**268,263**	**NI**			**ORPHAN**
92	NZ_CP049807.1	A1254	pA1254_1	37,834	RepM_Acin	cl45741	WP_167564445.1	ORPHAN
93	NZ_CP049808.1	A1254	pA1254_2	35,317	Rep_3	pfam01051	WP_167564503.1	ORPHAN
94	NZ_CP049809.1	A1254	pA1254_3	11,248	RepM_Acin	cl45741	WP_005133531.1	ORPHAN
95	NZ_CP049810.1	A1254	pA1254_4	11,269	RepM_Acin	cl45741	WP_005804946.1	ORPHAN
96	**NZ_CP054138.1**	**JXA13**	**pHNJXA13-1**	**206,931**	**NI**			**ORPHAN**
97	NZ_CP069498.1	FDAARGOS_1217	unnamed2	128,321	RepM_Acin	cl45741	WP_000818856.1	ORPHAN
98	NZ_CP069539.1	FDAARGOS_1214	unnamed2	4,178	RepM_Acin	cl45741	WP_016803254.1	ORPHAN
99	NZ_CP077241.1	FDAARGOS_1399	unnamed3	8,485	RepM_Acin	cl45741	WP_216972120.1	ORPHAN
100	NZ_CP077307.1	FDAARGOS_1396	unnamed4	5,594	NI			ORPHAN
101	NZ_CP084923.1	CEP14	pCEP14_02	58,452	RepM_Acin	cI45741	WP_005804946.1	ORPHAN
					RepM_Acin	cI45741	WP_005804946.1	
					RepM_Acin	cI45741	WP_005804946.1	
					RepM_Acin	cI45741	WP_005065049.1	
					RepM_Acin	cI45741	WP_005065049.1	
102	NZ_CP084924.1	CEP14	pCEP14_03	89,672	RepM_Acin	cl45741	WP_004843977.1	ORPHAN
					CyRepA1	cl26703, cl28734	WP_226789443.1	
103	**NZ_CP087717.1**	**AP2044**	**pAP2044-1**	**283,349**	**NI**			**ORPHAN**
104	**NZ_CP095410.1**	**TCM**	**pTCM-3**	**8,505**	**Rep_3**	**pfam01051**	**WP_004728629.1**	**ORPHAN**
105	NZ_CP107290.1	BM4623	p1	9,811	RepM_Acin	cI45741	WP_187406175.1	ORPHAN
106	NZ_CP123768.1	AP8900	pAP8900-3	12,558	RepM_Acin	cI45741	WP_199953184.1	ORPHAN
107	NZ_CP069541.1	FDAARGOS_1214	unnamed4	4,546	NI			ORPHAN
108	**NZ_MDIM02000005.1**	**UKK-0548**	**p5_UKK-0548**	**11,025**	**RepM_Acin**	**cl45741**	**WP_171258818.1**	**ORPHAN**
109	NZ_CP139250.1	MCR8900	pApiMCR8900a	7,614	RepM_Acin	cl45741	WP_000845851.1	ORPHAN
110	NZ_CP139252.1	MCR53	pApiMCR53c	23,644	RepM_Acin	cl45741	WP_069122403.1	ORPHAN
111	NZ_CP139253.1	MCR53	pApiMCR53b	4,208	RepM_Acin	cl45741	WP_320562525.1	ORPHAN
112	NZ_CP139254.1	MCR53	pApiMCR53a	3,916	NI			ORPHAN
113	NZ_CP139257.1	MCR16048	pApiMCR16048a	8,123	RepM_Acin	cl45741	WP_026441267.1	ORPHAN
114	NZ_CP139259.1	HUM3	pApiHUM3c	138,669	RepM_Acin	cl45741	WP_002046604.1	ORPHAN
115	NZ_CP139261.1	HUM3	pApiHUM3a	9,973	RepM_Acin	cl45741	WP_199982916.1	ORPHAN
116	NZ_CP139263.1	HUM1	pApiHUM1c	98,706	RepM_Acin	cl45741	WP_240296649.1	ORPHAN
117	NZ_CP139264.1	HUM1	pApiHUM1b	22,476	RepM_Acin	cl45741	WP_005804946.1	ORPHAN
					RepM_Acin	cl45741	WP_032066855.1	
118	NZ_CP139265.1	HUM1	pApiHUM1a	8,928	RepM_Acin	cl45741	WP_004896921.1	ORPHAN
119	NZ_CP139268.1	HUM14	pApiHUM14b	5,882	RepM_Acin	cl45741	WP_104039705.1	ORPHAN
120	NZ_CP139269.1	HUM14	pApiHUM14a	5,168	RepM_Acin	cl45741	WP_005065049.1	ORPHAN
121	NZ_CP139271.1	HCG62	pApiHCG62d	100,717	RepM_Acin	cl45741	WP_000818856.1	ORPHAN
122	NZ_CP139272.1	HCG62	pApiHCG62c	57,394	Rep_3-DUF5710	pfam01051, cl44662	WP_034700330.1	ORPHAN
123	NZ_CP139274.1	HCG62	pApiHCG62a	5,688	RepM_Acin	cl45741	WP_001208778.1	ORPHAN
124	NZ_CP139278.1	AN37	pApiAN37d	10,491	RepM_Acin	cl45741	WP_006582659.1	ORPHAN
125	NZ_CP139279.1	AN37	pApiAN37c	5,061	RepM_Acin	cl45741	WP_004698334.1	ORPHAN
126	NZ_CP139280.1	AN37	pApiAN37b	4,741	Replicase-PriCT	cl03886, cl07362	WP_320561151.1	ORPHAN
127	NZ_CP139281.1	AN37	pApiAN37a	3,964	Rep_1	cl02412	WP_086230553.1	ORPHAN
128	**NZ_CP139283.1**	**AE13**	**pApiAE13c**	**202,864**	**NI**			**ORPHAN**
129	NZ_CP139284.1	AE13	pApiAE13b	10,109	RepM_Acin	cl45741	WP_032066855.1	ORPHAN
130	NZ_CP139285.1	AE13	pApiAE13a	4,280	RepM_Acin	cl45741	WP_032859646.1	ORPHAN
131	NZ_CP139288.1	564 U	pApi564 Ub	64,705	RepM_Acin	cl45741	WP_002046604.1	ORPHAN
132	NZ_CP139289.1	564 U	pApi564 Ua	11,158	RepM_Acin	cl45741	WP_005065049.1	ORPHAN
133	NZ_CP139357.1	A45P	pApiA45Pc	154,609	NI			ORPHAN
134	NZ_CP139358.1	A45P	pApiA45Pb	11,667	RepM_Acin	cl45741	WP_048766123.1	ORPHAN
135	NZ_CP139359.1	A45P	pApiA45Pa	10,369	RepM_Acin	cl45742	WP_005244554.1	ORPHAN

Accession numbers of the plasmid DNA sequences, their names and sizes, description of the Rep proteins regarding to their protein domains, and if they belong or not (ORPHAN) to a plasmid lineage (PLP). Plasmids marked in bold letters carry antibiotic resistant genes.

### Virulence genes

To identify genes involved in virulence, we searched the Virulence Factor Database using the protein sequences encoded in the *A. pittii* strict core genome as a query. Surprisingly, only 15 of these proteins matched this database (Supplementary Table S3A). These genes are involved in immune modulation, biofilm formation, adherence, effector delivery, and regulation. We performed the same analysis using the *A. pittii* proteins encoded in the accessory genes as a query, and we found 81 matches with the VFDB (Supplementary Table S3B).

### The *A. pittii* plasmids

In the collection of high-quality *A. pittii* genome sequences, we found 135 plasmids with a wide size range, ranging from 2,295 bp to 283,349 bp. To evaluate their relationships, we grouped the plasmids according to their average nucleotide identities (ANI). With this strategy, we identified 17 plasmid lineages (PLP_1 to PLP_17). PLP_1, the largest lineage, contained 13 plasmids with sizes ranging from 39.5 to 48.5 kb. However, nine were isolated from diverse German locations between 2011 and 2014. The second-largest lineage, PLP_2, contained 11 plasmids, and PLP_3 had six members. The remaining plasmid lineages contained two or three members. However, 70 (51.4%) of these plasmids were orphans (Table 2).

The 135 *A. pittii* plasmids had, as a group, 6,882 genes; however, 4,673 (67.9%) of them encoded proteins annotated as hypothetical. These plasmids also possessed 665 transposase genes. We identified members of the 21 transposase families. The family with the greatest number of members was the IS3 family, with 227 members, followed by the IS5 family (144 members) and the IS6 family (71 members). This observation suggests that transposable elements have played a crucial role in the transfer of antibiotic resistance genes between plasmids and between plasmids and chromosomes.

The diversity of replication-initiation proteins encoded by the *A. pittii* plasmid was limited. Of the 111 Rep proteins identified in the plasmid collection, 86 belonged to the RepM_Acin superfamily (cl45741) according to the Conserved Domain Database classification (Lu et al., 2020). Interestingly, all plasmids encoding this Rep protein were adjacent to or near a gene or relics of a gene encoding an uncharacterized protein but were annotated as a *plasmid replication DNA-binding protein* (rep_pAB02_ORF2 superfamily). The next most abundant proteins, with 14 representatives, were Rep proteins with two protein domains: Rep_3 and DUF5710. Additionally, we found three Rep proteins having only a Rep_3 domain, two with a Rep_1 domain, and two containing the CyRepA1 domain. Finally, we detected two Rep proteins, one with an II domain and the other with Replicase-PriCT-HTH-23 domains. Nevertheless, we could not identify genes encoding replication initiator proteins in 33 (24.2%) plasmids from our collection (Table 2). Among the plasmids with no identifiable Rep genes were all members of the PLP_1, PLP_4, PLP_13, and PLP_15 plasmid lineages and 12 orphan plasmids.

Plasmids belonging to the same plasmid lineage encoded identical Rep proteins; for example, members of the PLP_2 plasmid lineage encoded Rep proteins with a protein identification number (protein_id) WP_000064928.1, or members of the PLP_3 lineage had Rep genes encoding proteins with the same id (WP_000064928.1). Similarly, PLP_5 contained Rep genes encoding WP_002046604.1 proteins. This situation is not rare in plasmids, as this has been described for many *A. baumannii* plasmids (Salgado-Camargo et al., 2020). Six of the *A. pittii* plasmids possessed genes encoding more than one Rep protein, which were usually not identical (different protein_id). The only exception was plasmid pCEP14_02, which encoded five Rep proteins with two different protein_ids (WP_005804946.1 and WP_005065049.1) (Table 2).

To evaluate the potential host range of the *A. pittii* plasmids, we searched for identical Rep proteins encoded by other plasmids. Our results, summarized in Supplementary Table S4, suggested that the host range of the *A. pittii* plasmids varied widely; some seemed to have a very narrow host range because we found only identical proteins encoded in other *A. pittii* plasmids. This was the case for the plasmids pIEC338SC2, pIEC338SC3, pA1254_1, and pApW20-3. In contrast, other plasmids appeared to be capable of replicating in an ample variety of *Acinetobacter* species, such as members of the PLP_2 and PLP_3 plasmid lineages. Moreover, we also found that some plasmids, such as the members of PLP_5, PLP_9, and PLP_14, shared identical Rep proteins with plasmids belonging to other genera, such as *Flavobacterium johnsoniae, Salmonella enterica,* and *Klebsiella pneumoniae.*

For the same purpose, we used the 135 *A. pittii* plasmids under study as queries in BLASTn searches, looking for plasmids with a DNA sequence identity equal to or greater than 95% and a query coverage of at least 75% in other *Acinetobacter* species. We found that 19 *A. pittii* plasmids were closely related to other *Acinetobacter* species, including *A. cumulans, A. chinensis, A. nosocomialis, A. baumannii, A. ursingii, A. wuhouensis, A. defluvii,* and *A. septicus.* Interestingly, 18 of the 19 plasmids that matched the plasmids of other *Acinetobacter* species carried antibiotic-resistance genes (Table 3). This observation suggested that non-*baumannii Acinetobacter* species are important reservoirs of antibiotic-resistance genes.

**Table 3:** *A. pittii* plasmids host-range.

	Accession_number_query	Plasmid_name_query	Accession_number_target	Plasmid_name_target	Q_Cov. (%)	ID (%)	Target_species
1	NZ_AGFH01000030.1	pAB_D499	CP035935.1	pNDM1_060092	97	99.98	*A. cumulans*
			CP032132.1	pNDM1_010005	81	99.99	*A. chinensis*
2	NZ_CM001802.1	pMX1	CP035935.1	pNDM1_060092	100	100	*A. cumulans*
			CP032132.1	pNDM1_010005	83	99.96	*A. chinensis*
3	NZ_MDHV02000003.1	pGIM1_UKK-0265	CP035935.1	pNDM1_060092	83	99.98	*A. cumulans*
4	NZ_MDHX02000003.1	pNDM1_UKK-0432	CP035935.1	pNDM1_060092	97	99.92	*A. cumulans*
			CP032132.1	pNDM1_010005	81	99.91	*A. chinensis*
5	NZ_MDIR02000003.1	pGIM1_UKK-0553	CP035935.1	pNDM1_060092	83	99.99	*A. cumulans*
6	NZ_MDIS02000003.1	pGIM1_UKK-0554	CP035935.1	pNDM1_060092	85	99.98	*A. cumulans*
7	NZ_MDIB02000003.1	pNDM1_UKK-0538	CP035935.1	pNDM1_060092	100	99.92	*A. cumulans*
			CP032132.1	pNDM1_010005	83	100	*A. chinensis*
8	NZ_MDIU02000005.1	pGIM1_UKK-0556	CP035935.1	pNDM1_060092	82	99.98	*A. cumulans*
9	NZ_MDIC02000004.1	pNDM1_UKK-0539	CP035935.1	pNDM1_060092	100	99.99	*A. cumulans*
			CP032132.1	pNDM1_010005	84	99.87	*A. chinensis*
10	NZ_MDIK02000003.1	pVIM2_UKK-0547	CP035935.1	pNDM1_060092	80	99.85	*A. cumulans*
11	NZ_MDIM02000003.1	pVIM2_UKK-0548	CP035935.1	pNDM1_060092	85	99.98	*A. cumulans*
12	NZ_MDID02000002.1	pNDM1_UKK-0540	CP035935.1	pNDM1_060092	100	99.99	*A. cumulans*
13	NZ_MDIT02000009.1	pGIM1_UKK-0555	CP035935.1	pNDM1_060092	85	99.98	*A. cumulans*
14	NZ_CP027659.1	pApW20-1	CP050433.1	pPM194229_1	75	99.99	*A. baumannii*
15	NZ_CP029006.1	pAbW39-1	CP076397.1	p2S8–227-229 k	77	99.94	*A. nosocomialis*
			CP087313.1	p1OC059	77	99.96	*A. baumannii*
16	NZ_CP095411.1	CP068183.1	CP068183.1	unnamed3	90%	98.89%	*A. ursingii*
17	NZ_MDIK02000007.1	p7_UKK-0547	CP068183.1	unnamed3	90%	96.92%	*A. ursingii*
18	NZ_CP042368.1	pC54_004	CP029390.1	p2_010030	75%	99.45	*A. defluvii*
			CP031712.1	p4_010060	75%	99.49%	*A. wuhouensis*
19	NZ_CP042365.1	pC54_001	CP029396.2	pOXA58_010030	84%	99.69	*A. defluvii*
			CP079899.1	unnamed	81%	98.92%	*A. septicus*

To evaluate the potential host-range of the *A. pittii* plasmids, we used as queries, in BLAST to searches, the 135 plasmids under analysis against the nr GenBank database. The column marked “Plasmid_name_query” lists these plasmids. Potential hosts were considering those *Acinetobacter* species with a plasmid that made a hit with any of the queries with a sequence identity of at least 95% and a coverage of equal to or higher than 75% (last column at right). The column named “Plasmid_name_target” lists the plasmids that made a hit with these cutoff values. Plasmids marked in blue carry antibiotic resistance genes.

*A. pittii* plasmids are crucial in disseminating antibiotic resistance genes; 30.1% of the plasmids studied here carried genes involved in antibiotic resistance. All members of lineages PLP_1, PLP_5, PLP_11, PLP_12, PLP_13, and PLP_15 carried antibiotic resistance genes, but orphan plasmids also functioned in the spread of antibiotic resistance genes, considering that 16 of the 70 orphan plasmids carried genes involved in antibiotic resistance (Table 2). Importantly, some plasmids, such as those belonging to the plasmid lineages PLP_5, PLP_9, and PLP_14, had a very wide potential host range that included other pathogens, such as *A. baumannii, K. pneumoniae,* and *S. enterica.*

Table 4 lists all the antibiotic resistance genes found in the *A. pittii* plasmids. Genes involved in aminoglycoside resistance were the most common. However, genes conferring resistance to carbapenems, such as *bla*NDM1, *bla*NDM44, *bla*OXA58, *bla*OXA72, *bla*GIM1, and *blaVIM1,* were also found. The number of antibiotic resistance genes encoded in plasmids varied widely; some, such as pAB_D499 or p7_UKK-0548, carried only one antibiotic resistance gene, while others contained up to 12 different genes involved in antibiotic resistance, such as plasmid pC54_001. Frequently, antibiotic resistance genes were near IS sequences, indicating the role of transposable elements in the dispersion of antibiotic resistance.

**Table 4:** *A. pittii* plasmids carrying antibiotic resistance genes.

	Accession_number	Strain	Plasmid_name	Plasmid_size (bp)	Best_Hit_ARO	Drug class	Resistance mechanism
1	NZ_AGFH01000030.1	D499	pAB_D499	47,101	APH(3′)-VIa	aminoglycoside antibiotic	antibiotic inactivation
					NDM-1	carbapenem; cephalosporin; cephamycin; penam; penem	antibiotic inactivation
2	NZ_CM001802.1	XM1570	pXM1	47,274	APH(3′)-VIa	aminoglycoside antibiotic	antibiotic inactivation
					NDM-1	carbapenem; cephalosporin; cephamycin; penam; penem	antibiotic inactivation
3	NZ_CP014478.1	AP_882	pNDM-AP_882	146,597	AAC(3)-IId	aminoglycoside antibiotic	antibiotic inactivation
					sul2	sulfonamide antibiotic	antibiotic target replacement
					APH(3″)-Ib	aminoglycoside antibiotic	antibiotic inactivation
					APH(6)-Id	aminoglycoside antibiotic	antibiotic inactivation
					NDM-1	carbapenem; cephalosporin; cephamycin; penam	antibiotic inactivation
4	NZ_CP014479.1	AP_882	pOXA58-AP_882	36,862	msrE	macrolide antibiotic; streptogramin antibiotic	antibiotic target protection
					mphE	macrolide antibiotic	antibiotic inactivation
					OXA-58	carbapenem; cephalosporin; penam	antibiotic inactivation
5	NZ_CP015146.1	IEC338SC	pIEC338SCOX	10,498	OXA-72	carbapenem; cephalosporin; penam	antibiotic inactivation
6	NZ_CP018910.1	XJ88	unnamed1	9,501	OXA-72	carbapenem; cephalosporin; penam	antibiotic inactivation
7	NZ_CP026086.2	WCHAP005069	pOXA58_005069	112,436	OXA-58	carbapenem; cephalosporin; penam	antibiotic inactivation
					AAC(3)-IId	aminoglycoside antibiotic	antibiotic inactivation
					sul2	sulfonamide antibiotic	antibiotic target replacement
					APH(3″)-Ib	aminoglycoside antibiotic	antibiotic inactivation
					APH(6)-Id	aminoglycoside antibiotic	antibiotic inactivation
					APH(3′)-VIa	aminoglycoside antibiotic	antibiotic inactivation
					APH(6)-Id	aminoglycoside antibiotic	antibiotic inactivation
					mphE	macrolide antibiotic	antibiotic inactivation
					msrE	macrolide antibiotic; streptogramin antibiotic	antibiotic target protection
8	NZ_CP027249.2	WCHAP100004	pOXA58_100004	105,591	mphE	macrolide antibiotic	antibiotic inactivation
					msrE	macrolide antibiotic; streptogramin antibiotic	antibiotic target protection
					OXA-58	carbapenem; cephalosporin; penam	antibiotic inactivation
					AAC(3)-IId	aminoglycoside antibiotic	antibiotic inactivation
					floR	phenicol antibiotic	antibiotic efflux
					sul2	sulfonamide antibiotic	antibiotic target replacement
					APH(3″)-Ib	aminoglycoside antibiotic	antibiotic inactivation
					APH(3′)-VIb	aminoglycoside antibiotic	antibiotic inactivation
					PER-1	monobactam; carbapenem; cephalosporin; penam; penem	antibiotic inactivation
					APH(3″)-Ib	aminoglycoside antibiotic	antibiotic inactivation
					APH(6)-Id	aminoglycoside antibiotic	antibiotic inactivation
9	NZ_CP027253.1	WCHAP100020	pOXA58_100020	24,018	OXA-58	carbapenem; cephalosporin; penam	antibiotic inactivation
					msrE	macrolide antibiotic; streptogramin antibiotic	antibiotic target protection
					mphE	macrolide antibiotic	antibiotic inactivation
10	NZ_CP027659.1	Ap-W20	pApW20-1	237,519	sul2	sulfonamide antibiotic	antibiotic target replacement
					APH(6)-Id	aminoglycoside antibiotic	antibiotic inactivation
					APH(3″)-Ib	aminoglycoside antibiotic	antibiotic inactivation
11	NZ_CP028573.2	WCHAP005046	pOXA58_005046	61,751	msrE	macrolide antibiotic; streptogramin antibiotic	antibiotic target protection
					mphE	macrolide antibiotic	antibiotic inactivation
					OXA-58	carbapenem; cephalosporin; penam	antibiotic inactivation
12	NZ_CP029006.1	AbW39	pAbW39-1	262,817	sul2	sulfonamide antibiotic	antibiotic target replacement
					APH(6)-Id	aminoglycoside antibiotic	antibiotic inactivation
					APH(3″)-Ib	aminoglycoside antibiotic	antibiotic inactivation
13	NZ_CP029611.1	ST220	unnamed	84,302	sul2	sulfonamide antibiotic	antibiotic target replacement
14	NZ_CP040912.1	AB17H194	pAB17H194–1	88,002	sul2	sulfonamide antibiotic	antibiotic target replacement
					APH(3′)-Ia	aminoglycoside antibiotic	antibiotic inactivation
					mphE	macrolide antibiotic	antibiotic inactivation
					msrE	macrolide antibiotic; streptogramin antibiotic	antibiotic target protection
					TEM-2	monobactam; cephalosporin; penam; penem	antibiotic inactivation
					AAC(3)-IIe	aminoglycoside antibiotic	antibiotic inactivation
					APH(6)-Id	aminoglycoside antibiotic	antibiotic inactivation
					dfrA1	diaminopyrimidine antibiotic	antibiotic target replacement
					APH(6)-Id	aminoglycoside antibiotic	antibiotic inactivation
					tet(X5)	tetracycline antibiotic	antibiotic inactivation
					APH(3″)-Ib	aminoglycoside antibiotic	antibiotic inactivation
15	NZ_CP042365.1	C54	pC54_001	256,887	sul2	sulfonamide antibiotic	antibiotic target replacement
					msrE	macrolide antibiotic; streptogramin antibiotic	antibiotic target protection
					mphE	macrolide antibiotic	antibiotic inactivation
					floR	phenicol antibiotic	antibiotic efflux
					sul1	sulfonamide antibiotic	antibiotic target replacement
					catB3	phenicol antibiotic	antibiotic inactivation
					AAC(6′)-Ib4	aminoglycoside antibiotic	antibiotic inactivation
					IMP-26	carbapenem; cephalosporin; cephamycin; penam; penem	antibiotic inactivation
					dfrA19	diaminopyrimidine antibiotic	antibiotic target replacement
					OXA-58	carbapenem; cephalosporin; penam	antibiotic inactivation
					AAC(3)-IId	aminoglycoside antibiotic	antibiotic inactivation
					APH(3′)-VIa	aminoglycoside antibiotic	antibiotic inactivation
16	NZ_CP043053.1	AP43	pAP43-OXA58-NDM1	268,263	AAC(3)-IId	aminoglycoside antibiotic	antibiotic inactivation
					OXA-58	carbapenem; cephalosporin; penam	antibiotic inactivation
					msrE	macrolide antibiotic; streptogramin antibiotic	antibiotic target protection
					mphE	macrolide antibiotic	antibiotic inactivation
					floR	phenicol antibiotic	antibiotic efflux
					NDM-1	carbapenem; cephalosporin; cephamycin; penam	antibiotic inactivation
					adeF	fluoroquinolone antibiotic; tetracycline antibiotic	antibiotic efflux
					APH(3′)-VIa	aminoglycoside antibiotic	antibiotic inactivation
17	NZ_CP054138.1	JXA13	pHNJXA13-1	206,931	APH(3′)-VIa	aminoglycoside antibiotic	antibiotic inactivation
					AAC(3)-IId	aminoglycoside antibiotic	antibiotic inactivation
					mphE	macrolide antibiotic	antibiotic inactivation
					msrE	macrolide antibiotic; streptogramin antibiotic	antibiotic target protection
					tet(39)	tetracycline antibiotic	antibiotic inactivation
					tet(X3)	glycylcycline; tetracycline antibiotic	antibiotic inactivation
					sul2	sulfonamide antibiotic	antibiotic target replacement
					NDM-1	carbapenem; cephalosporin; cephamycin; penam	antibiotic inactivation
18	NZ_CP087717.1	AP2044	pAP2044-1	206,931	NDM-1	carbapenem; cephalosporin; cephamycin; penam	antibiotic inactivation
					APH(3′)-VIa	aminoglycoside antibiotic	antibiotic inactivation
					AAC(3)-IId	aminoglycoside antibiotic	antibiotic inactivation
					adeF	fluoroquinolone antibiotic; tetracycline antibiotic	antibiotic efflux
19	NZ_CP087718.1	AP2044	pAP2044-2	43,577	msrE	macrolide antibiotic; streptogramin antibiotic	antibiotic target protection
					mphE	macrolide antibiotic	antibiotic inactivation
					tet(39)	tetracycline antibiotic	antibiotic inactivation
					sul2	sulfonamide antibiotic	antibiotic target replacement
					APH(3″)-Ib	aminoglycoside antibiotic	antibiotic inactivation
					APH(6)-Id	aminoglycoside antibiotic	antibiotic inactivation
20	NZ_CP095408.1	TCM	pTCM1	84,108	sul2	sulfonamide antibiotic	antibiotic target replacement
21	NZ_CP095410.1	TCM	pTCM3	8,505	mphE	macrolide antibiotic	antibiotic inactivation
					msrE	macrolide antibiotic; streptogramin antibiotic	antibiotic target protection
22	NZ_CP095411.1	TCM	pTCM4	6,078	ANT(2″)-Ia	aminoglycoside antibiotic	antibiotic inactivation
23	NZ_CP123766.1	AP8900	pAP8900-1	86,394	sul2	sulfonamide antibiotic	antibiotic target replacement
24	NZ_CP123767.1	AP8900	pAP8900-2	43,600	msrE	macrolide antibiotic; streptogramin antibiotic	antibiotic target protection
					sul2	sulfonamide antibiotic	antibiotic target replacement
					tet(39)	tetracycline antibiotic	antibiotic inactivation
					APH(3″)-Ib	aminoglycoside antibiotic	antibiotic inactivation
					mphE	macrolide antibiotic	antibiotic inactivation
					APH(6)-Id	aminoglycoside antibiotic	antibiotic inactivation
25	NZ_MDHV02000003.1	UKK-0265	pGIM1_UKK-0265	39,562	APH(3′)-VIa	aminoglycoside antibiotic	antibiotic inactivation
					sul1	sulfonamide antibiotic	antibiotic target replacement
					ANT(2″)-Ia	aminoglycoside antibiotic	antibiotic inactivation
					GIM-1	monobactam; carbapenem; cephalosporin; cephamycin; penam; penem	antibiotic inactivation
26	NZ_MDHX02000003.1	UKK-0432	pNDM1_UKK-0432	48,580	APH(3′)-VIa	aminoglycoside antibiotic	antibiotic inactivation
					NDM-1	carbapenem; cephalosporin; cephamycin; penam	
27	NZ_MDIB02000003.1	UKK-0538	pNDM1_UKK-0538	47,278	APH(3′)-VIa	aminoglycoside antibiotic	antibiotic inactivation
					APH(3′)-Ia	aminoglycoside antibiotic	
					NDM-1	carbapenem; cephalosporin; cephamycin; penam; penem	antibiotic inactivation
28	NZ_MDIC02000004.1	UKK-0539	pNDM1_UKK-0539,	49,842	APH(3′)-VIa	aminoglycoside antibiotic	antibiotic inactivation
					NDM-44	carbapenem; cephalosporin; cephamycin; penam; penem	antibiotic inactivation
29	NZ_MDID02000002.1	UKK-0540	pNDM1_UKK-0540	46,164	APH(3′)-VIa	aminoglycoside antibiotic	antibiotic inactivation
30	NZ_MDIK02000003.1	UKK-0547	pVIM2_UKK-0547	46,530	APH(3′)-VIa	aminoglycoside antibiotic	antibiotic inactivation
					VIM-2	carbapenem; cephalosporin; cephamycin; penam; penem	antibiotic inactivation
					AAC(6′)-Ib9	aminoglycoside antibiotic	antibiotic inactivation
					sul1	sulfonamide antibiotic	antibiotic target replacement
31	NZ_MDIK02000005.1	UKK-0547	p5_UKK-0547	8,804	APH(3′)-Ia	aminoglycoside antibiotic	antibiotic inactivation
32	NZ_MDIK02000007.1	UKK-0547	p7_UKK-0547	6,078	ANT(2″)-Ia	aminoglycoside antibiotic	antibiotic inactivation
33	NZ_MDIM02000003.1	UKK-0548	pVIM2_UKK-0548	45,234	sul1	sulfonamide antibiotic	antibiotic target replacement
					AAC(6′)-Ib3	aminoglycoside antibiotic	antibiotic inactivation
					VIM-2	carbapenem; cephalosporin; cephamycin; penam; penem	antibiotic inactivation
					APH(3′)-VIa	aminoglycoside antibiotic	antibiotic inactivation
34	NZ_MDIM02000005.1	UKK-0548	p5_UKK-0548	11,025	tet(39)	tetracycline antibiotic	
35	NZ_MDIM02000007.1	UKK-0548	p7_UKK-0548	8,804	APH(3′)-Ia	aminoglycoside antibiotic	antibiotic inactivation
36	NZ_MDIR02000003.1	UKK-0553	pGIM1_UKK-0553	43,536	APH(3′)-VIa	aminoglycoside antibiotic	antibiotic inactivation
					sul1	sulfonamide antibiotic	antibiotic target replacement
					ANT(2″)-Ia	aminoglycoside antibiotic	antibiotic inactivation
					GIM-1	monobactam; carbapenem; cephalosporin; cephamycin; penam; penem	antibiotic inactivation
37	NZ_MDIS02000003.1	UKK-0554	pGIM1_UKK-0554	42,583	APH(3′)-VIa	aminoglycoside antibiotic	antibiotic inactivation
					sul1	sulfonamide antibiotic	antibiotic target replacement
					ANT(2″)-Ia	aminoglycoside antibiotic	antibiotic inactivation
					GIM-1	monobactam; carbapenem; cephalosporin; cephamycin; penam; penem	antibiotic inactivation
38	NZ_MDIT02000009.1	UKK-0555	pGIM1_UKK-0555	42,583	APH(3′)-VIa	aminoglycoside antibiotic	antibiotic inactivation
					sul1	sulfonamide antibiotic	antibiotic target replacement
					ANT(2″)-Ia	aminoglycoside antibiotic	antibiotic inactivation
					GIM-1	monobactam; carbapenem; cephalosporin; cephamycin; penam; penem	antibiotic inactivation
39	NZ_MDIU02000005.1	UKK-0556	pGIM1_UKK-0556	43,772	APH(3′)-VIa	aminoglycoside antibiotic	antibiotic inactivation
					sul1	sulfonamide antibiotic	antibiotic target replacement
					ANT(2″)-Ia	aminoglycoside antibiotic	antibiotic inactivation
					GIM-1	monobactam; carbapenem; cephalosporin; cephamycin; penam; penem	antibiotic inactivation
40	NZ_CP139283.1	AE13	pApiAE13c	202,864	APH(6)-Id	aminoglycoside antibiotic	antibiotic inactivation
					APH(3″)-Ib (4 copies)	aminoglycoside antibiotic	antibiotic inactivation
					sul2	sulfonamide antibiotic	antibiotic target replacement
41	NZ_CP107290.1	BM4623	p1	9,811	tet(39)	tetracycline antibiotic	antibiotic inactivation
42	NZ_CP139252	MCR53	pApiMCR53c	23,644	OXA-72	carbapenem; cephalosporin; penam	antibiotic inactivation

GenBank accession numbers of the DNA plasmid sequences, strains harboring the plasmid, plasmid sizes, the antibiotic resistance gene that they carry and the drug class which they belong.

Analysis of the distribution of the plasmids grouped into lineages (PLP_1 to PLP_17) revealed that members of the same lineage were scattered throughout the core genome phylogenetic tree (Figure 2) (Table 2). This was evidence that these genetic elements were subject to horizontal transfer events. However, none had a complete set of genes required for conjugation. The plasmid with the greatest number of genes involved in the synthesis of a Type IV secretion system (T4SS) required for conjugation was the plasmid pNDM1_UKK-0432, which contained 10 of the 12 core proteins that Gram-negative bacteria require for this process (Costa et al., 2021). Thirty-seven plasmids had MobA/MobL-type proteins, suggesting that they were potentially mobilizable.

Interestingly, a search of the gene annotations of the *A. pittii* genomes revealed that most did not contain genes encoding Type IV secretion systems. A few of these genes, except those mentioned above, contained, at most, 5 genes involved in the synthesis and function of this secretion apparatus.

### Phylogenomic analysis at the local level

To examine the population dynamics more closely at the local level, we sequenced and analyzed the genomes of 29 clinical isolates obtained from Third-level hospitals: Two are in Mexico City, three are in different Mexican states (provinces), and one in Honduras. We also sequenced a strain isolated from a red-lored parrot and two *A. pittii* strains obtained from a Panamanian frogs, one of the genomes sequences was completed and closed (Cevallos et al., 2022; Bello-López et al., 2024). Depending on their position in a core-genome phylogenetic tree, we obtained the complete and closed genome sequences of 14 isolates representing different clades. The ANI values of 28 genome sequences, when contrasted with the *A. pittii* type strain, were greater than 95%, confirming that they belonged to *A. pittii*. However, four strains, MCR8900, AbaK, HUM6, and AN51, exhibited ANI values in a range from 92 to 95%. Among named *Acinetobacter* species, *A. pittii* had the closest ANI to these four strains, indicating that they did not belong to *A. pittii* as a species but were closely related to it. For this reason, we will call them *A. pittii-*like, provisionally (A. Nemec, personal communication).

**Figure 3:**
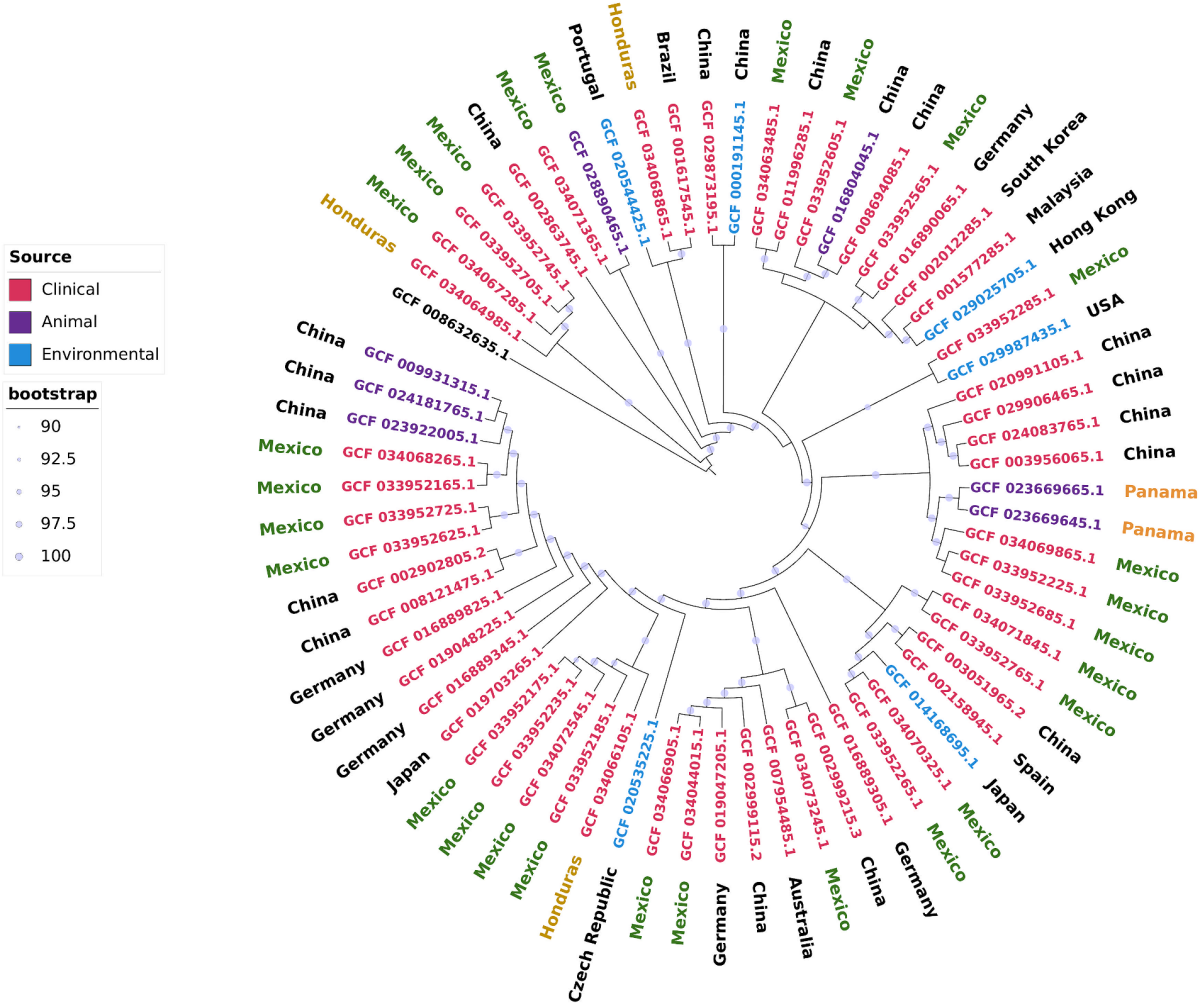
Core genome maximum likelihood phylogenetic tree including the complete and closed *A. pittii* genomes downloaded from NCBI and those sequenced in this work. *A. baumannii* (CP046654.1) was used as outgroup.

To evaluate the relationships and phylogeography of the strains from Mexico and Honduras, we constructed a maximum likelihood phylogenetic tree of the core genome (Figure 3). We included 36 complete and closed *A. pittii* genome sequences in this tree. These genome sequences were obtained from strains isolated in 14 countries and from environmental, animal, and human (clinical) sources.

First, we observed that all the isolates from Mexico were dispersed throughout the phylogenetic tree. Moreover, isolates from the same hospital generally did not cluster in the same clade. The exceptions were strains AN37, AN38, and AN42, which were isolated from the same patient over almost 2 years. These observations indicated that the Mexican hospitals included in our study suffer several independent introductions and that several genetically different isolates are circulating within the country. The second significant observation was that Mexican isolates were closely related to isolates from the Far East (continental China, Taiwan, and Malaysia). These results confirmed only the observation that *A. pittii* isolates were subject to intercontinental dissemination, as stated above. The third observation was that the hospital-acquired isolates were closely linked to environmental isolates. For example, strain WP2-W18-ESBL-11 isolated from wastewater was closely related to the Mexican hospital-acquired isolates 564 U and 539 U, and in the same sense, strain PHEA-2 obtained from wastewater was also close to INC1094, a hospital-acquired isolate. The fourth observation was that some isolates from Mexico and other hospital-acquired isolates were linked to those obtained from animals, including some that were sick. For instance, strain 978-A_19 was isolated from a red-lored parrot with pneumonia and kept in captivity in Mexico City, and the Chinese strain Ap-W20 was obtained from a sick fish from a farm (*Megalobrama amblycephala*) (Li et al., 2017; Bello-López et al., 2024). The four strains close to *A. pittii* were grouped in the same clade, and they likely deserved to be considered new species; however, better characterization is needed before a name can be given to this possible new species.

An analysis of the antibiotic resistance genes of the strains sequenced in this work revealed that all the isolates possessed intrinsic *blaOXA* genes belonging to the *bla*OXA*213* family. Thirteen also had a *bla*ADC-25-like gene. One strain, HUM8, had three *blaOXA* genes; one was a member of the *blaOXA213* family, and the other two were part of the *blaOXA143* family. Strain MCR53 had a plasmid encoding the *blaOXA72* gene. Finally, AE13 had six plasmid-encoded genes related to antibiotic resistance. Five were related to aminoglycoside resistance, and another, *sul2*, was related to sulfonamide resistance (Table 5).

**Table 5:** Antibiotic resistance genes present on the strains sequenced in this work.

	Strain	Accession_num	*bla*OXA-143_family	*bla*OXA-213_family	*bla*OXA-24/40_family	*bla*ADC-25_family	aminoglycoside_res.	Sulfonamide_res.
1	34H	GCF_033952765.1		blaOXA-506 (100)		blaADC-25 (92.93)		
2	539 U	GCF_033952265.1		blaOXA-417 (100)		blaADC-25 (91.76)		
3	564 U	GCF_034070325.1		blaOXA-417 (100)		blaADC-25 (91.76)		
4	A45P	GCF_023669645.1		blaOXA-564 (99.51)				
				blaOXA-506 (99.51)				
5	A47H	GCF_023669665.1		blaOXA-564 (99.51)				
				blaOXA-506 (99.51)				
6	AbaK	GCF_034067285.1		blaOXA-270 (97.81)		blaADC-25 (95.92)		
7	AE13	GCF_034073245.1		blaOXA-500 (100)			aph(6)-Id (100)	sul2
							aph(3″)-Ib (100)	
							aph(3″)-Ib (99.88)	
							aph(3″)-Ib (99.88)	
							aph(3″)-Ib (99.88)	
8	AN37	GCF_034072545.1		blaOXA-500 (100)				
9	AN38	GCF_033952175.1		blaOXA-500 (100)				
10	AN42	GCF_033952235.1		blaOXA-500 (100)				
11	AN51	GCF_033952745.1		blaOXA-270 (99.64)		blaADC-25 (90.71)		
12	HCG138	GCF_034071365.1		**blaOXA-1164**		**blaADC-286**		
13	HCG18	GCF_034063485.1		**blaOXA-1165**		**blaADC-287**		
				blaOXA-533 (97.69)				
14	HCG62	GCF_034071845.1		blaOXA-506 (100)		blaADC-25 (91.93)		
15	HUM1	GCF_034068265.1		blaOXA-500 (99.88)				
16	HUM10	GCF_033952725.1		blaOXA-500 (99.88)				
17	HUM11	GCF_033952685.1		blaOXA-506 (99.51)				
				blaOXA-564 (99.51)				
18	HUM12	GCF_033952225.1		blaOXA-506 (99.51)				
				blaOXA-564 (99.51)				
19	HUM13	GCF_033952285.1		blaOXA-506 (98.54)		blaADC-25 (91.33)		
				blaOXA-564 (98.54)				
				blaOXA-526 (98.54)				
20	HUM14	GCF_034069865.1		blaOXA-564 (99.51)				
				blaOXA-506 (99.51)				
21	HUM2	GCF_033952165.1		blaOXA-500 (99.88)				
22	HUM3	GCF_034066905.1		blaOXA-272 (99.76)				
23	HUM4	GCF_033952185.1		blaOXA-500 (100)				
24	HUM5	GCF_034044015.1		blaOXA-272 (99.76)				
25	HUM6	GCF_033952705.1		blaOXA-270 (99.51)		blaADC-25 (90.97)		
26	HUM7	GCF_033952625.1		blaOXA-500 (99.88)				
27	HUM8	GCF_033952605.1	blaOXA-255 (99.64)	blaOXA-272 (99.66)		blaADC-25 (92.1)		
			blaOXA-499 (99.64)					
28	INC1094	GCF_033952565.1		blaOXA-506 (98.18)				
				blaOXA-564 (98.18)				
29	MCR16048	GCF_034066105.1		blaOXA-500 (100)				
30	MCR53	GCF_034068865.1		**blaOXA-1166**	blaOXA72 (100)	**blaADC-287**		
31	MCR8900	GCF_034064985.1		**blaOXA-270 (93.92)**		blaADC-25 (90.29)		
32	978-A_19	GCF_028890465.1		blaOXA-564 (99.15)		**blaADC-292**		
				blaOXA-506 (99.15)				

The name of the strains, their GenBank accession numbers and the antibiotic resistance genes are listed. Between parentheses the percentage of identity against the closest match. Strains marked in grey are new *Acinetobacter* species close to *A. pittii* (*pittii*-like). The rest of the strains are *A. pittii*. New antibiotic resistance alleles are in bold letters. The new alleles were deposited at the beta-Lactamase_DataBase (http://bldb.eu/BLDB.php?prot=D#OXA).

**Figure 4:**
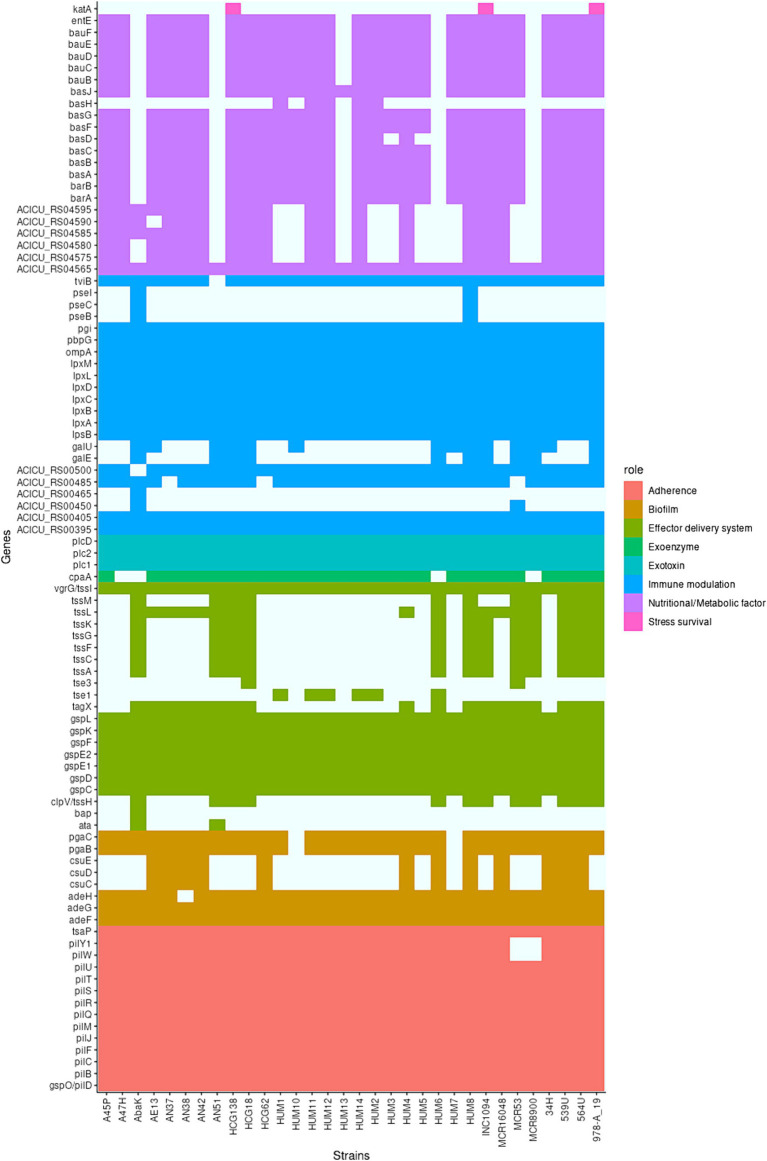
Virulence genes present in the strains sequenced in this work. On the left, virulence genes that are found in each of the strains sequenced in this work. On the right, their potential role in virulence. A colored box indicates the presence of the gene.

As a group, the genes of these strains, matched 92 virulence genes (from the VFDB database). All shared a common set of 39 virulence genes, but the number of virulence genes per strain varied widely. Noteworthy, the four “*pittii*-like” strains has less virulence related genes (Figure 4).

## Discussion

When we started the analysis of *A. pittii* from a genomic perspective, we thought we would find a structured population where strains coexisting in a given site (geography), environment (i.e., water or soil), or specific hosts (like plants or animals) were going to share alleles and/or specific genes, which in some ways were going to twin them. However, in contrast to what was initially thought, this study demonstrated that *A. pittii* isolates obtained from both hospital environments and non-clinical settings frequently share the same clade in a phylogenetic tree constructed with the core genome. Additionally, we showed that *A. pittii* isolates from different geographical regions, even from different continents, may be closely related in the same clade in a core genome-based phylogenetic tree. We also showed that strains obtained in the same hospital might not be closely related, suggesting that hospitals may have suffered multiple independent introductions of genetically distinct groups of this pathogen. These observations indicated that *A. pittii* lacks evident migratory barriers. In this context, it is important to note that the constant immigration of new genes or alleles into a given population delays or prevents the population from structuring.

The possible migration vectors can be very varied. In the first place, as previously documented, traveling has proven to be an effective route for introducing multidrug-resistant bacteria from one country to another (Birgand et al., 2014; Higgins et al., 2021). Secondly, since it has been possible to isolate *A. pittii* from a wide range of animals including mammals, amphibians, birds, and fish, some of whom have migratory habits, they could also easily function as migration vectors (Li et al., 2017; Morakchi et al., 2017; Wang et al., 2020; Ying et al., 2020; Cevallos et al., 2022; Leelapsawas et al., 2022). Thirdly, *A. pittii* has also been isolated from aquatic environments like freshwater and seawater, so these means could facilitate the migration of members of this species from one site to another (Kong et al., 2021; Koskeroglu et al., 2023). These observations raise the possibility of genetic exchange between *A. pittii* strains from different geographical origins.

Plasmids are efficient vectors for disseminating virulence and antibiotic-resistance genes among bacterial strains within the same species and between different species and genera (San Millan, 2018; MacLean and San Millan, 2019). The capacity of these plasmids to transfer genes will depend mainly on whether they are conjugative or mobilizable, as well as their host range. Here, we observe that a considerable proportion of *A. pittii* plasmids (51.4%) are classified as orphans, suggesting a potentially wide diversity of plasmids within this species. On the other hand, the rest of the plasmids belong to a few lineages, most composed of only two or three members. This situation differs from what is observed in *A. baumannii,* where most plasmids belong to a very limited number of lineages (Salgado-Camargo et al., 2020). This disparity may be attributed to more frequent plasmid exchange between *A. baumannii* strains belonging to international clones already adapted to the hospital environment than those isolated from non-hospital environments.

A close examination of Rep proteins present in *A. pittii* plasmids suggests that their host range spectrum varies considerably. While some plasmids appear to have a marked specificity, others can replicate in a wide range of *Acinetobacter* species, including *A. baumannii.* This analysis also indicates that some of these plasmids may replicate in species outside the *Acinetobacter* genus (see Supplementary Table S4). However, a recent study revealed that the most common Rep proteins in *A. baumannii* belong to the Rep_3 superfamily. In contrast, the sample of *A. pittii* plasmids studied here exhibits Rep genes frequently associated with the RepM_Acin superfamily. These observations suggest that, although many *A. pittii* plasmids may theoretically replicate in *A. baumannii*, the exchange of plasmids between these two species remains limited. If exchanges were more frequent, a similar distribution of different Rep families between both species would be expected. Nevertheless, such an exchange is likely to occur at low frequency. By analyzing the DNA sequences of the 135 *A. pittii* plasmids using BLAST in NCBI, we found that some show significant similarities to plasmids from other *Acinetobacter* species, including *A. baumannii.*

Our analysis also revealed that 28.6% of *A. pittii* plasmids carry antibiotic-resistance genes, but no evidence of virulence-related genes was found. Furthermore, none of the plasmids encoded a complete set of genes for a type IV secretion system, indicating they are not self-transmissible. However, members of each of the plasmid lineages of *A. pittii* are dispersed throughout the phylogenetic tree constructed with the core genome (Figure 1), suggesting they are horizontally transferred with the help of other plasmids (mobilization), or using different mechanisms such as transformation, transduction, or through outer membrane vesicles (OMVs) (Rumbo et al., 2011; Chatterjee et al., 2017).

Acquiring plasmids with antibiotic-resistance genes will be crucial in the initial differentiation between environmental isolates and those leading to hospital-acquired infections. This differentiation will reduce the genetic diversity of nosocomial isolates and enhance their fitness in the hospital environment. Other characteristics that will be important in hospital adaptation and that some *A. pittii* strains already possess include forming biofilms and desiccation resistance. Therefore, we suggest that this organism is taking its first steps to become an emerging nosocomial pathogen and, thus, could be an excellent model for studying this process (Bravo et al., 2018; Chapartegui-González et al., 2022; Wunderlich et al., 2023).

As shown above, *A. pittii* strains of environmental, animal, or plant origin may be closely related to strains isolated from hospital patients. A first interpretation of this association is that non-hospital strains may be pathogenic if they find an immunocompromised right host and an invading route, as has already been observed for other emerging pathogens. The problem is that although the two strains are closely related by core genome, the number and gene content of their accessory genomes may differ, and these differences may include those genes encoding virulence factors. In fact, we found in the accessory genome genes encoding a phospholipase C (an exotoxin), metabolic factors that allow its persistence in the host, such as genes involved in the synthesis of Acinetobactin (Song and Kim, 2020), genes important in the adhesion to solid surfaces as the type IV *pilus* (Vo et al., 2023), in the formation of biofilms like the chaperone-usher *pili* (Csu) (Ahmad et al., 2023), or genes encoding the synthesis machinery of capsular polysaccharides. It should be noted that the ability of *A. baumannii* and *A. pittii* to adhere to solid surfaces and form biofilms is not only important in the infection process, but these characteristics could also explain their persistence in the hospital environment (Rajangam and Narasimhan, 2024).

Another way to interpret these observations is that all strains of *A. pittii* contain a platform of genes that could allow it to infect an immunocompromised patient if a route of infection exists, but for this to occur efficiently, an additional set of virulence genes must be acquired from other strains. Thus, for example, if a strain can infect an animal, it must acquire additional genes to adapt to humans. Given the differences in the number of genes between two given strains, an extensive system of horizontal gene transfer is needed, which cannot be sustained by conjugation alone. As such, transformation could be a more efficient way for this phenomenon to occur. Extensive experimental evidence is needed to support the hypotheses we put forward here.

## Materials and methods

### Genome sequence collection

In this work, we used two genome sequence sources: first, we downloaded all genome sequences available in the RefSeq and GenBank databases (NCBI) in May 2022. The quality of the genome sequences was evaluated regarding their completeness and degree of contamination with CheckM (Parks et al., 2015). Assemblies with less than 95% of completeness or possessing a contamination rate higher than 5% were eliminated. To evaluate if the downloaded genomes were correctly identified as *A. pittii*, we calculated the pairwise average nucleotide Average Nucleotide Identity (ANI) of all genome sequences against the type strain (ATCC 19004, accession number: GCF_000369045.1) with pyANI v0.2.9 (Pritchard et al., 2016). Genome sequences with an ANI value less than 95% against the type strain were eliminated from our collection, taking into consideration that the recommended species delineation threshold of ANI is 95–96%. After these filters, we kept a study set of 352 *A. pittii* genome sequences (Supplementary Table S1). Second, to increase the diversity of our genome collection, we obtained the complete genome sequences of 26 Mexican *A. pittii* isolates from six different hospitals located in different regions of the country (Table 1). Ten of these genomes were closed and circularized. Also, we also obtained the genome sequences of two Panamanian frogs, one of them was circularized and closed (Cevallos et al., 2022). Additionally, we did the same procedure with the genome sequences of three Honduran hospital-acquired *A. pittii* isolates.

### Genome sequence determinations

Genomic DNA of all strains was extracted from overnight cultures grown overnight at 37°C and 250 rpm in 3 mL of Luria–Bertani broth, using the Genomic DNA purification kit (Thermo-Fisher) following the manufacturer’s instructions, with a small modification: samples were treated with RNAse (10 ng/mL) (Thermo-Fisher) at 37°C, 30 min prior final DNA precipitation. Draft genome sequences of the Mexican strains were obtained by sequencing short read libraries (2 × 150 bp) with the BGISEQ-2000 platform at the Beijing Genomics Institute, China. The Honduran strain’s genome sequences were obtained with an Illumina MiSeq platform with 2 × 300 bp paired-end reads at the Instituto Nacional de Medicina Genómica (INMEGEN, México). Trim Galore v0.6.4, developed by Babrahan Bioinformatics, was used to remove adapters and quality trimming of the sequencing reads.

Genome short-read assemblies were constructed using three algorithms: Velvet 1.2.10 (Zerbino and Birney, 2008), SPAdes 3.9.0 (Bankevich et al., 2012), and ABySS 2.0.1 (Jackman et al., 2017), and with several kmers. The best assembly obtained with each program was selected to obtain a merged and optimized assembly for each strain using Metassembler 1.5 (Wences and Schatz, 2015). Additionally, 14 *A. pittii* isolates were selected to close and circularize their genome sequences, using an Oxford-Nanopore device (PrometION). Oxford-Nanopore libraries were constructed and sequenced at the Beijing Genomic Institute (China). Adapter sequences were removed using Porechop 0.2.4 and base calling was performed with Guppy 5.1.13v using the high-accuracy base-calling mode. Hybrid assemblies were obtained utilizing Unicycler 0.4.8 (Wick et al., 2017).

Assembly statistics were calculated with Quast (v5.0.2) (Gurevich et al., 2013). The completeness and degree of contamination of these assemblies were evaluated with CheckM (Parks et al., 2015). Finally, genome sequences were annotated with the NCBI Prokaryotic Genome Annotation Pipeline (Tatusova et al., 2016). GenBank accession numbers are shown in Table 1.

### Virulence and antibiotic-resistance genes identification

To identify genes involved in antibiotic resistance, we consulted the Comprehensive Antibiotic Resistance Database CARD 3.1.4 with RGI (Resistant Gene Identifier) and selected perfect or strict hits (Alcock et al., 2020). For plasmids, we also search the ResFinder database (v4.4.2) at https://www.genomicepidemiology.org (Florensa et al., 2022). The virulence genes were identified by consulting through BLASTp, the Virulence Factor Database (setA) (VFDB), asking for matches with an E value of 0 and a sequence identity of at least 80% (Liu et al., 2022). Only representative genes linked to experimentally validated Virulence Factors are included in the VFDB setA, or core dataset.

### Phylogenetic tree construction

The first phylogenetic tree constructed includes all of the strains in our genome collection. The second tree considers the *A. pittii* Refseq complete genome sequences from NCBI and the strains from Mexico and Honduras that were sequenced by us. To do this, the strict core genome of the strains was obtained using Roary (v3.13.0) (Page et al., 2015), and with these data, maximum likelihood phylogenetic trees were constructed using IQ-TREE (v2.1.4-beta) with a TIM2 + F + R10 substitution mode. We used as an outgroup the genome sequence of *A. baumannii* ATCC19606 (CP046654.1). Also, we constructed a phylogenetic tree based on the presence/absence of genes of the *A. pittii* pangenome, using IQ-TREE (v2.1.4-beta) for binary data analysis. Trees were visualized and annotated with iTOL (Letunic and Bork, 2021). To evaluate Single Nucleotide Variants (SNVs), a consensus core-genome of 279 aligned clusters, without evidence of recombination, was obtained using GET_HOMOLOGUES and GET_PHYLOMARKERS (Contreras-Moreira and Vinuesa, 2013; Vinuesa et al., 2018). The SNVs present in the alignments were then evaluated with show-snps which is a utility MUMmer 3.0 software (Kurtz et al., 2004).

### Plasmid sequence analysis

In this work, we analyzed the DNA sequences of 135 plasmids obtained from the complete and closed genomes of *A. pittii* (Table 2). To evaluate their relationships, we first calculated the average nucleotide sequences of all potential plasmid pairs. Then, we sorted the plasmids in two stages: in the first one, the plasmids were grouped based on their nucleotide sequence identities (≥ 95%) with a sequence coverage of 75% or higher. In the second stage, we incorporated new members into a specific the group if the new plasmid had a sequence identity of ≥95% and a sequence coverage of ≥75% with at least one member of the group.

The plasmid replication initiation (Rep) proteins were identified by analyzing the genome annotations provided by NCBI and classifying them according to their protein domains using the CD-search tool at NCBI. To evaluate the potential host range of the plasmids, we search for identical Rep proteins present in other plasmids in other species. We considered potential host species those that shared the same Rep in one of their plasmids.

To identify plasmids similar to those of *A. pittii* in other *Acinetobacter* species, we used as probes, in BLASTn searches at NCBI, the DNA sequences of the 135 *A. pittii* plasmids described in this work. In these searches, we considered those plasmids with a DNA sequence identity of at least 95% and coverage equal to or greater than 70%.

## Data availability statement

The datasets presented in this study can be found in online repositories. The names of the repository/repositories and accession number(s) can be found in the article/Supplementary material.

## Author contributions

EB-L: Data curation, Formal analysis, Investigation, Writing – review & editing. AE-M: Data curation, Formal analysis, Investigation, Methodology, Visualization, Writing – review & editing. GG: Data curation, Formal analysis, Writing – review & editing. AC-C: Data curation, Formal analysis, Writing – review & editing. EG-G: Data curation, Formal analysis, Writing – review & editing. RH-C: Data curation, Formal analysis, Writing – review & editing. PZ: Data curation, Formal analysis, Writing – review & editing. RM-O: Data curation, Formal analysis, Writing – review & editing. PV: Data curation, Formal analysis, Writing – review & editing. JX-C: Data curation, Formal analysis, Writing – review & editing. MC: Conceptualization, Formal analysis, Funding acquisition, Investigation, Project administration, Resources, Writing – original draft, Writing – review & editing.
