# Supplementary material for: Acinetobacter pittii: the emergence of a hospital-acquired pathogen analyzed from the genomic perspective
Source: Front Microbiol. 2024 Jun 26;15:1412775. doi: 10.3389/fmicb.2024.1412775 (PMC11233732; doi:10.3389/fmicb.2024.1412775)
Supplement: Supplementary file 1 [file Data_Sheet_1.PDF]

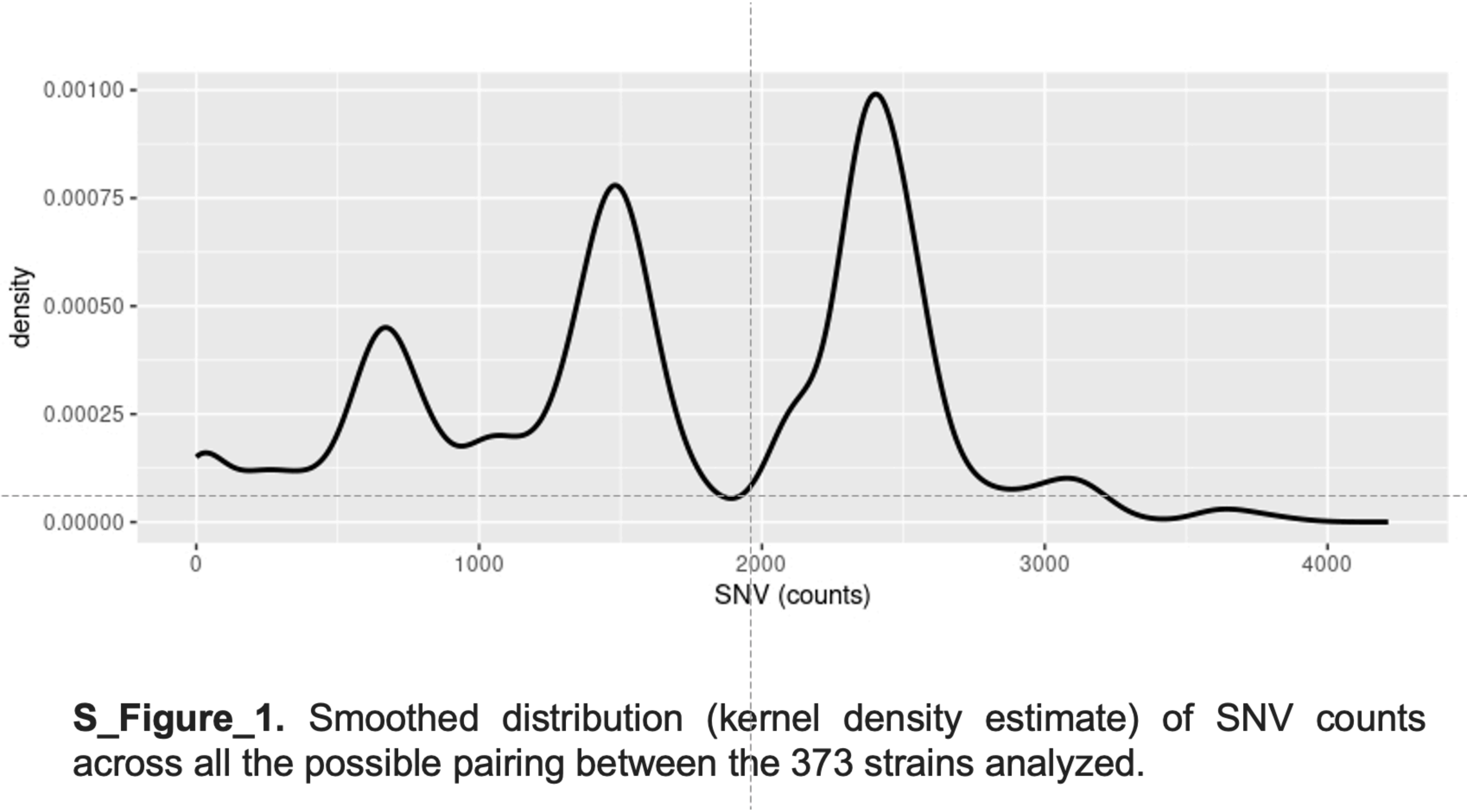

S_Figure_1. Smoothed distribution (kernel density estimate) of SNV counts across all the possible pairing between the 373 strains analyzed.
