# Supplementary material for: Acinetobacter pittii: the emergence of a hospital-acquired pathogen analyzed from the genomic perspective
Source: Front Microbiol. 2024 Jun 26;15:1412775. doi: 10.3389/fmicb.2024.1412775 (PMC11233732; doi:10.3389/fmicb.2024.1412775)
Supplement: Supplementary file 5 [file Data_Sheet_5.pdf]

Accession_num. NCBI	strain	country
GCF_022449145.1	17014	China
GCF_022449185.1	18052	China
GCF_022449155.1	18069	China
GCF_022449025.1	19101	China
GCF_020544425.1	32292	Portugal
GCF_022214835.1	2018EL-00061	USA
GCF_022214765.1	2018EL-00062	USA
GCF_026420025.1	2018EL-00068	USA
GCF_022397275.1	2018EL-00069	USA
GCF_022397255.1	2018EL-00070	USA
GCF_022397135.1	2018EL-00073	USA
GCF_001056355.1	332_ABCC	USA
GCF_000529235.1	42F	Colombia
GCF_900495015.1	4300STDY7045719	Thailand
GCF_900496515.1	4300STDY7045887	Thailand
GCF_026622735.1	A-136	China
GCF_026622515.1	A-140	China
GCF_026622715.1	A-223	China
GCF_026622495.1	A-225	China
GCF_026622795.1	A-32	China
GCF_026622685.1	A-340	China
GCF_026622945.1	A-37	China
GCF_026622595.1	A-6	China
GCF_026622835.1	A-8	China
GCF_011996285.1	A1254	China
GCF_022937775.1	A154	China
GCF_022937665.1	A160	China
GCF_008694085.1	AB17H194	China
GCF_001432715.1	ABBL005	USA
GCF_001432775.1	ABBL010	USA
GCF_001432435.1	ABBL015	USA
GCF_001432835.1	ABBL019	USA
GCF_001432635.1	ABBL024	USA
GCF_001432935.1	ABBL031	USA
GCF_001433125.1	ABBL033	USA
GCF_001433185.1	ABBL042	USA
GCF_001433295.1	ABBL046	USA
GCF_001433225.1	ABBL047	USA
GCF_001414705.1	ABBL064	USA
GCF_001414725.1	ABBL065	USA
GCF_001415105.1	ABBL074	USA
GCF_001415185.1	ABBL077	USA
GCF_001415175.1	ABBL078	USA
GCF_001415935.1	ABBL096	USA
GCF_001415585.1	ABBL103	USA
GCF_001416065.1	ABBL120	USA
GCF_001416185.1	ABBL135	USA
GCF_001416335.1	ABBL148	USA
GCF_001936495.1	ABC	India
GCF_002161985.1	ABUH403	USA
GCF_002014165.1	ABUH411	USA
GCF_024181765.1	AbW39	China
GCF_017162055.1	AC15140	Malaysia
GCF_017161945.1	AC1525	Malaysia
GCF_017162015.1	AC1555	Malaysia
GCF_017162025.1	AC1566	Malaysia
GCF_017161985.1	AC1597	Malaysia
GCF_017162095.1	AC1640	Malaysia
GCF_017161975.1	AC1649	Malaysia
GCF_017162065.1	AC1822	Malaysia
GCF_904887025.1	Aci00748	Germany
GCF_904887545.1	Aci00901	Germany
GCF_904887685.1	Aci00905	Germany
GCF_016502795.1	ACIN00171	USA
GCF_016502765.1	ACIN00178	USA
GCF_016508425.1	ACIN00188	USA
GCF_016502645.1	ACIN00192	USA
GCF_016502655.1	ACIN00195	USA
GCF_016502575.1	ACIN00202	USA
GCF_016502535.1	ACIN00216	USA
GCF_016502395.1	ACIN00238	USA
GCF_016502385.1	ACIN00239	USA
GCF_016506275.1	ACIN00247	USA
GCF_016506165.1	ACIN00255	USA
GCF_016508345.1	ACIN00256	USA
GCF_009646055.1	AMA10	Argentina
GCF_009645895.1	AMA2	Argentina
GCF_000369025.1	ANC_3678	Czech Republic
GCF_001577285.1	AP_882	Malaysia
GCF_018109465.1	Ap-D8	China
GCF_023922005.1	Ap-W20	China
GCF_006351765.1	AP007	Canada
GCF_016535745.1	AP1	Thailand
GCF_020991105.1	AP2044	China
GCF_016535875.1	AP273	Thailand
GCF_008121475.1	AP43	China
GCF_016522335.1	AP436	Thailand
GCF_019459025.1	Ap45	Switzerland
GCF_016535865.1	AP56	Thailand
GCF_016535885.1	AP864	Thailand
GCF_029906465.1	AP8900	China
GCF_016623485.1	AP984	Thailand
GCF_002137365.1	ARLG-1763	USA
GCF_002137175.1	ARLG-1765	USA
GCF_002137935.1	ARLG-1773	USA
GCF_002136575.1	ARLG-1799	USA
GCF_002137635.1	ARLG-1805	USA
GCF_002137415.1	ARLG-1812	USA
GCF_002137885.1	ARLG-1825	USA
GCF_002137125.1	ARLG-1832	USA
GCF_002136755.1	ARLG-1861	USA
GCF_002137855.1	ARLG-1870	USA
GCF_002138095.1	ARLG-1945	USA
GCF_002138225.1	ARLG-1949	USA
GCF_002137095.1	ARLG-1951	USA
GCF_002137145.1	ARLG-1952	USA
GCF_002137115.1	ARLG-1953	USA
GCF_002138235.1	ARLG-1954	USA
GCF_002137805.1	ARLG-1958	USA
GCF_002137615.1	ARLG-1960	USA
GCF_002144015.1	ARLG1764	USA
GCF_002144035.1	ARLG1768	USA
GCF_002143025.1	ARLG1771	USA
GCF_002143415.1	ARLG1776	USA
GCF_002143135.1	ARLG1779	USA
GCF_002143175.1	ARLG1789	USA
GCF_002143255.1	ARLG1798	USA
GCF_002143515.1	ARLG1803	USA
GCF_002143505.1	ARLG1804	USA
GCF_002143595.1	ARLG1807	USA
GCF_002143895.1	ARLG1839	USA
GCF_002145325.1	ARLG1865	USA
GCF_002145265.1	ARLG1942	USA
GCF_002145315.1	ARLG1950	USA
GCF_002144875.1	ARLG1957	USA
GCF_002144885.1	ARLG1961	USA
GCF_010608795.1	AS012594	USA
GCF_010612505.1	AS012606	USA
GCF_018595725.1	B-3780	Russia
GCF_003954385.1	B8245	South Korea
GCF_029873195.1	BM4623	China
GCF_007954485.1	C54	Australia
GCF_002761185.1	CEB-Ap	Portugal
GCF_020535225.1	CEP14	Czech Republic
GCF_000369045.1	CIP_70.29	NA
GCF_024390955.1	CIP70.29	USA
GCF_019264765.1	Colony142	Thailand
GCF_929606335.1	cpe037	United Kingdom
GCF_000800555.1	CR12-42	Australia
GCF_000230465.1	D499	China
GCF_007680445.1	DE0027	USA
GCF_007679545.1	DE0091	USA
GCF_000836015.1	DSM_25618	NA
GCF_017166625.1	F8_7S_12B	International Space Station
GCF_017166585.1	F8_7S_13B	International Space Station
GCF_017166605.1	F8_7S_14B	International Space Station
GCF_017166565.1	F8_7S_15B	International Space Station
GCF_017166525.1	F8_7S_16B	International Space Station
GCF_017166545.1	F8_7S_17B	International Space Station
GCF_017166485.1	F8_7S_18B	International Space Station
GCF_017166385.1	F8_7S_4B	International Space Station
GCF_017166365.1	F8_7S_5B	International Space Station
GCF_017166275.1	F8_7S_6P	International Space Station
GCF_017166265.1	F8_7S_7B	International Space Station
GCF_017166225.1	F8_8S_11B	International Space Station
GCF_017166205.1	F8_8S_12B	International Space Station
GCF_017166125.1	F8_8S_2P	International Space Station
GCF_017166095.1	F8_8S_6P	International Space Station
GCF_001684275.1	FC8876	Brazil
GCF_016889825.1	FDAARGOS_1214	Germany
GCF_016889345.1	FDAARGOS_1215	Germany
GCF_016890065.1	FDAARGOS_1216	Germany
GCF_016889305.1	FDAARGOS_1217	Germany
GCF_019048225.1	FDAARGOS_1396	Germany: Braunschweig
GCF_019047205.1	FDAARGOS_1399	Germany
GCF_029841065.1	GD03848	Pakistan
GCF_029837025.1	GD04049	USA
GCF_002927935.1	HDV312665	Honduras
GCF_026704985.1	HNA001	China
GCF_026704485.1	HNA046	China
GCF_026704365.1	HNA052	China
GCF_026704185.1	HNA066	China
GCF_016811255.1	HU_AC	USA
GCF_002158945.1	HUMV-6483	Spain
GCF_001617545.1	IEC338SC	Brazil
GCF_002740775.1	IHIT24944	Germany
GCF_002740745.1	IHIT29469	Germany
GCF_002740785.1	IHIT29592	Germany
GCF_002740735.1	IHIT32473	Germany
GCF_002740805.1	IHIT32685	Germany
GCF_001743435.1	IIF1SW-P1	NA
GCF_013449825.1	IIF1SW-P2	International Space Station
GCF_013450185.1	IIF1SW-P3	International Space Station
GCF_013449855.1	IIF1SW-P4	International Space Station
GCF_013450165.1	IIF1SW-P5	International Space Station
GCF_025515655.1	INTEC_OZDC10	Dominican Republic
GCF_001698835.1	IPK_TSA6.1	South Korea
GCF_016804045.1	JXA13	China
GCF_001707545.1	KCJK1729	USA
GCF_003053325.1	KCJK7889	USA
GCF_013344885.1	KCJK8569	USA
GCF_023721105.1	LAM11	Benin
GCF_001005885.2	LC510	USA
GCF_023605635.1	Ltt201	China
GCF_023605235.1	Ltt241	China
GCF_902386175.1	MGYG-HGUT-02346	USA
GCF_021460885.1	MIN-007	Bangladesh
GCF_029025705.1	ML4	Hong Kong
GCF_000931895.1	NBRC_110504	Japan
GCF_000949735.1	NBRC_110505	Japan
GCF_000949755.1	NBRC_110506	Japan
GCF_000949775.1	NBRC_110507	Japan
GCF_000931915.1	NBRC_110508	Japan
GCF_000949795.1	NBRC_110509	Japan
GCF_000934125.1	NBRC_110510	Japan
GCF_006538605.1	NBRC_110514	Japan
GCF_009931315.1	NQ-003	China
GCF_003569665.1	OCU_Ac12	Japan
GCF_019703265.1	OCU_Ac17	Japan
GCF_003569475.1	OCU_Ac3	Japan
GCF_003569515.1	OCU_Ac5	Japan
GCF_003569535.1	OCU_Ac6	Japan
GCF_024649535.1	OLA15	Benin
GCF_024580095.1	PAC15	Benin
GCF_000191145.1	PHEA-2	China
GCF_021385025.1	R1009-1	China
GCF_021386775.1	R550	China
GCF_021385975.1	R820	China
GCF_029270005.1	S-30	India
GCF_009822185.1	SCsl25	NA
GCF_023002065.1	SK070	Thailand
GCF_015694545.1	SQ079	China
GCF_001706055.1	St-10592-91	Germany
GCF_001706045.1	St-11469-92	Germany
GCF_001706435.1	St-12275-92	Germany
GCF_001706275.1	St-12537-91	Germany
GCF_001706715.1	St-12828-92	Germany
GCF_001706645.1	St-14379-92	Germany
GCF_001706365.1	St-14569-91	Germany
GCF_001706505.1	St-15559-91	Germany
GCF_001706665.1	St-15639-91	Germany
GCF_001706525.1	St-17942-91	Germany
GCF_001706065.1	St-19650-90	Germany
GCF_001706585.1	St-20772-90	Germany
GCF_003956065.1	ST220	China
GCF_018422535.1	Survcare344	Germany
GCF_012910685.1	sw-1	Pacific Ocean
GCF_000805655.1	T167	Thailand
GCF_021384265.1	T550-1	China
GCF_021388455.1	T820	China
GCF_021388445.1	T822-1	China
GCF_024083765.1	TCM	China
GCF_001716955.1	TCM156	China
GCF_001716945.1	TCM178	China
GCF_001716935.1	TCM292	China
GCF_000817365.1	TE2	India
GCF_003978965.1	TG22174	USA
GCF_003947895.1	TG22176	USA
GCF_003947695.1	TG29420	USA
GCF_003947915.1	TG29422	USA
GCF_003947955.1	TG31972	USA
GCF_003947975.1	TG31979	USA
GCF_003947655.1	TG31980	USA
GCF_003947985.1	TG40858	USA
GCF_003947705.1	TG41012	USA
GCF_003947725.1	TG41017	USA
GCF_003947765.1	TG41230	USA
GCF_003947995.1	TG41243	USA
GCF_003948045.1	TG41247	USA
GCF_003948075.1	TG41884	USA
GCF_000302375.1	TG6411	NA
GCF_003947755.1	TG91944	USA
GCF_003948335.1	TG91976	USA
GCF_021388055.1	TJ8-2	China
GCF_008580785.1	TUM16154	NA
GCF_008581325.1	TUM16165	NA
GCF_001706035.1	UKK-0145	Turkey
GCF_001706695.1	UKK-0245	Germany
GCF_001706115.1	UKK-0250	Germany
GCF_001706125.2	UKK-0265	Germany
GCF_001706565.1	UKK-0327	Germany
GCF_001706155.2	UKK-0432	Belgium
GCF_001706135.1	UKK-0520	Germany
GCF_001706195.1	UKK-0536	Germany
GCF_001706415.2	UKK-0538	Germany
GCF_001706555.2	UKK-0539	Germany
GCF_001713665.2	UKK-0540	Germany
GCF_001706575.1	UKK-0541	Germany
GCF_001706205.1	UKK-0542	Germany
GCF_001706635.1	UKK-0543	Germany
GCF_001706215.1	UKK-0544	Germany
GCF_001706225.1	UKK-0545	Germany
GCF_001713675.1	UKK-0546	Germany
GCF_001706735.2	UKK-0547	Germany
GCF_001706775.2	UKK-0548	Germany
GCF_001706425.1	UKK-0549	Germany
GCF_001706445.1	UKK-0550	Germany
GCF_001706285.1	UKK-0551	Germany
GCF_001706305.1	UKK-0552	Germany
GCF_001706315.2	UKK-0553	Germany
GCF_001706355.2	UKK-0554	Germany
GCF_001713685.2	UKK-0555	Germany
GCF_001706495.2	UKK-0556	Germany
GCF_900110525.1	UNC434CL69Tsu2S25	NA
GCF_026936235.1	VJP_2022a	USA
GCF_009887685.1	VNMU_150	Ukraine
GCF_000315155.1	WC-136	Irak
GCF_003051965.2	WCHAP005046	China
GCF_002902805.2	WCHAP005069	China
GCF_004209725.1	WCHAP100001	China
GCF_004209635.1	WCHAP100002	China
GCF_004209565.1	WCHAP100003	China
GCF_002999115.2	WCHAP100004	China
GCF_004209655.1	WCHAP100005	China
GCF_004209625.1	WCHAP100006	China
GCF_004209605.1	WCHAP100007	China
GCF_004209595.1	WCHAP100008	China
GCF_004208835.1	WCHAP100009	China
GCF_004209555.1	WCHAP100010	China
GCF_004209515.1	WCHAP100011	China
GCF_004209305.1	WCHAP100012	China
GCF_004209495.1	WCHAP100013	China
GCF_004209435.1	WCHAP100014	China
GCF_004209505.1	WCHAP100015	China
GCF_004209445.1	WCHAP100016	China
GCF_004296295.1	WCHAP100017	China
GCF_004209415.1	WCHAP100018	China
GCF_002999215.3	WCHAP100020	China
GCF_004209395.1	WCHAP100021	China
GCF_004209295.1	WCHAP100022	China
GCF_004209335.1	WCHAP100023	China
GCF_000746375.1	WP19	USA
GCF_014168695.1	WP2-W18-ESBL-11	Japan
GCF_025513075.1	WU_MDCI_Ap04	USA
GCF_025513035.1	WU_MDCI_Ap08	USA
GCF_025514035.1	WU_MDCI_Ap104	USA
GCF_025514055.1	WU_MDCI_Ap105	USA
GCF_025514095.1	WU_MDCI_Ap119	USA
GCF_025514135.1	WU_MDCI_Ap138	USA
GCF_025514155.1	WU_MDCI_Ap141	USA
GCF_025514175.1	WU_MDCI_Ap142	USA
GCF_025514215.1	WU_MDCI_Ap144	USA
GCF_025514295.1	WU_MDCI_Ap148	USA
GCF_025514395.1	WU_MDCI_Ap167	USA
GCF_025514455.1	WU_MDCI_Ap171	USA
GCF_025514475.1	WU_MDCI_Ap172	USA
GCF_025514535.1	WU_MDCI_Ap178	USA
GCF_025514595.1	WU_MDCI_Ap188	USA
GCF_025514615.1	WU_MDCI_Ap197	USA
GCF_025514695.1	WU_MDCI_Ap218	USA
GCF_025515035.1	WU_MDCI_Ap258	USA
GCF_025515135.1	WU_MDCI_Ap266	USA
GCF_025513635.1	WU_MDCI_Ap44	USA
GCF_025513565.1	WU_MDCI_Ap45	USA
GCF_025513735.1	WU_MDCI_Ap49	USA
GCF_025513765.1	WU_MDCI_Ap62	USA
GCF_025562415.1	WU_MDCI_Ap64	USA
GCF_025562375.1	WU_MDCI_Ap68	USA
GCF_025514225.1	WU_MDCI_Ap81	USA
GCF_002863745.1	XJ88	China
GCF_000341835.1	XM1570	China
GCF_002012285.1	YMC2010_8_T3	South Korea

S_Table_1. Accession numbers of the *A. pittii* genomes downloaded from GenBanK, the name of the strains, and their country of origin.
