# Supplementary material for: Acinetobacter pittii: the emergence of a hospital-acquired pathogen analyzed from the genomic perspective
Source: Front Microbiol. 2024 Jun 26;15:1412775. doi: 10.3389/fmicb.2024.1412775 (PMC11233732; doi:10.3389/fmicb.2024.1412775)
Supplement: Supplementary file 6 [file Data_Sheet_6.PDF]

Best_Hit_ARO	Cov. (%)	Ident. (%)	Gene Family
A. baumannii AbaF	100	99.07	major facilitator superfamily (MFS) antibiotic efflux pump
A. baumannii AbaQ	100	98.85	major facilitator superfamily (MFS) antibiotic efflux pump
A. baumannii AbaQ	100	99.77	major facilitator superfamily (MFS) antibiotic efflux pump
A. baumannii AmvA	100	96.14	major facilitator superfamily (MFS) antibiotic efflux pump
A. baumannii AmvA	100	95.12	major facilitator superfamily (MFS) antibiotic efflux pump
AAC(3)-IIc	107.34	96.69	AAC(3)
AAC(6')-31	95.93	100	AAC(6')
AAC(6')-Ib8	76.44	100	AAC(6')
AAC(6')-IIa	100	100	AAC(6')
AadA	103.04	99.62	ANT(3'')
AadA2	100	99.61	ANT(3'')
AadA5	100	100	ANT(3'')
AadT	100	99.21	major facilitator superfamily (MFS) antibiotic efflux pump
ADC-18	100	100	ADC beta-lactamase without carbapenemase activity
AdeL	101.78	97.32	resistance-nodulation-cell division (RND) antibiotic efflux pump
ANT(2'')-Ia	102.26	97.74	ANT(2'')
ANT(2'')-Ia	100	100	ANT(2'')
ANT(3'')-IIa	100	96.56	ANT(3'')
ANT(3'')-IIa	82.44	96.3	ANT(3'')
ANT(3'')-IIb	99.62	48.99	ANT(3'')
APH(3'')-Ib	106.74	99.25	APH(3'')
APH(3'')-Ib	100	99.63	APH(3'')
APH(3')-Ia	100	100	APH(3')
APH(3')-VIa	100	98.46	APH(3')
APH(3')-VIa	100	100	APH(3')
APH(3')-VIa	100	98.46	APH(3')
APH(3')-VIb	100	100	APH(3')
APH(6)-Id	100	99.64	APH(6)
Arr-2	100	100	rifampin ADP-ribosyltransferase (Arr)
CARB-16	100	100	CARB beta-lactamase
CARB-3	100	99.67	CARB beta-lactamase
CatB3	100	100	chloramphenicol acetyltransferase (CAT)
CmlA5	100	98.57	major facilitator superfamily (MFS) antibiotic efflux pump
CmlB1	100	100	major facilitator superfamily (MFS) antibiotic efflux pump
DfrA1	100	99.36	trimethoprim resistant dihydrofolate reductase dfr
DfrA10	100	100	trimethoprim resistant dihydrofolate reductase dfr
DfrA19	100	99.47	trimethoprim resistant dihydrofolate reductase dfr
FloR	100	99.75	major facilitator superfamily (MFS) antibiotic efflux pump
GIM-1	100	100	GIM beta-lactamase
IMP-14	100	100	IMP beta-lactamase
IMP-19	100	100	IMP beta-lactamase
IMP-4	100	100	IMP beta-lactamase
MCR-1.1	100	100	MCR phosphoethanolamine transferase
MphE	100	100	macrolide phosphotransferase (MPH)
MsrE	100	100	msr-type ABC-F protein
NDM-1	100	100	NDM beta-lactamase
OXA-10	100	99.62	OXA beta-lactamase; OXA-10-like beta-lactamase
OXA-21	100	100	OXA beta-lactamase; OXA-2-like beta-lactamase
OXA-23	100	100	OXA beta-lactamase; OXA-23-like beta-lactamase
OXA-499	100	100	OXA beta-lactamase; OXA-143-like beta-lactamase
OXA-500	100	100	OXA beta-lactamase; OXA-213-like beta-lactamase
OXA-506	100	100	OXA beta-lactamase; OXA-213-like beta-lactamase
OXA-542	100	100	OXA beta-lactamase
OXA-58	91.79	100	OXA beta-lactamase; OXA-58-like beta-lactamase
OXA-72	100	100	OXA beta-lactamase; OXA-24-like beta-lactamase
OXA-72	100	100	OXA beta-lactamase; OXA-24-like beta-lactamase
OXA-72	100	100	OXA beta-lactamase; OXA-24-like beta-lactamase
PER-1	100	100	PER beta-lactamase
QacEdelta1	100	100	major facilitator superfamily (MFS) antibiotic efflux pump
QacG	106.54	50	small multidrug resistance (SMR) antibiotic efflux pump
QnrVC6	100	100	quinolone resistance protein (qnr)
Sul1	100	100	sulfonamide resistant sul
Sul2	106.27	100	sulfonamide resistant sul
Sul2	100	100	sulfonamide resistant sul
TEM-2	100	100	TEM beta-lactamase
Tet(39)	100	100	major facilitator superfamily (MFS) antibiotic efflux pump
Tet(X)	100	99.48	tetracycline inactivation enzyme
Tet(X3)	100	100	tetracycline inactivation enzyme
Tet(X5)	100	100	tetracycline inactivation enzyme
VEB-21	100	100	VEB beta-lactamase
VIM-2	100	100	VIM beta-lactamase

S_Table_2. Antibiotic resistance protein found encoded in the A. pittii accessory genome in the CARD database. First column is the best hit in the Antibiotic Resistance Ontology, their sequence coverage and sequence identity with the best match in the database. The last column is the gene family of the best hit.
