# Supplementary material for: Acinetobacter pittii: the emergence of a hospital-acquired pathogen analyzed from the genomic perspective
Source: Front Microbiol. 2024 Jun 26;15:1412775. doi: 10.3389/fmicb.2024.1412775 (PMC11233732; doi:10.3389/fmicb.2024.1412775)
Supplement: Supplementary file 7 [file Data_Sheet_7.PDF]

VFDB_accession_number	Gene	Anotation	Role_in_virulence
VFG037720(gb|WP_001027056)	adeG	Cation/multidrug efflux pump [AdeFGH efflux pump]	Biofilm
VFG037747(gb|WP_001984639)	lpxM	Lysophospholipid acyltransferase family protein [LPS]	Immune modulation
VFG037762(gb|WP_000078878)	lpxL	Lauroyl acyltransferase [LPS]	Immune modulation
VFG037777(gb|WP_000867091)	lpsB	Glycosyltransferase family 4 protein [LPS]	Immune modulation
VFG037792(gb|WP_000064875)	lpxA	Acyl-ACP--UDP-N-acetylglucosamine O-acyltransferase [LPS]	Immune modulation
VFG037837(gb|WP_000868104)	lpxD	UDP-3-O-(3-hydroxymyristoyl)glucosamine N-acyltransferase [LPS]	Immune modulation
VFG038249(gb|WP_003384760)	pbpG	D-alanyl-D-alanine endopeptidase PBP7/8 [PbpG]	Immune modulation
VFG050313(gb|WP_004644147.1)	pilM	Pilus assembly protein PilM [TFP]	Adherence
VFG050400(gb|WP_000347039.1)	pilU	PilT/PilU family type 4a pilus ATPase [TFP]	Adherence
VFG050414(gb|WP_002027454.1)	pilF	Type IV pilus biogenesis/stability protein PilW [TFP]	Adherence
VFG050499(gb|WP_000755268.1)	tsaP	LysM peptidoglycan-binding domain-containing protein [TFP]	Adherence
VFG050699(gb|WP_000840548.1)	pilR	Sigma-54 dependent transcriptional regulator [TFP]	Adherence
VFG050757(gb|WP_001166319.1)	gspE1	General secretion pathway protein E [T2SS]	Effector delivery system
VFG050787(gb|WP_001112089.1)	gspF	General secretion pathway protein F [T2SS]	Effector delivery system
VFG050874(gb|WP_001989189.1)	gspL	General secretion pathway protein L [T2SS]	Effector delivery system

S_Table_3A. Virulence genes of *A. pittii*, belonging to the core genome, found in the VFDB database.
