# Supplementary material for: Acinetobacter pittii: the emergence of a hospital-acquired pathogen analyzed from the genomic perspective
Source: Front Microbiol. 2024 Jun 26;15:1412775. doi: 10.3389/fmicb.2024.1412775 (PMC11233732; doi:10.3389/fmicb.2024.1412775)
Supplement: Supplementary file 9 [file Data_Sheet_9.pdf]

VFDB_accession_number	Gene	Anotation	Role_in_virulence
VFG037176(gb|WP_001081735)	plc1	Phospholipase C [Phospholipase C]	Exotoxin
VFG037177(gb|WP_000632986)	plc2	Phospholipase C [Phospholipase C]	Exotoxin
VFG037203(gb|WP_000079188)	plcD	Phosphatidylserine/phosphatidylglycerophosphate/cardiolipin synthase [Phospholipase D]	Exotoxin
VFG037218(gb|WP_000983821)	basJ	Acinetobactin biosynthesis protein basj [Acinetobactin]	Nutritional/Metabolic factor
VFG037260(gb|WP_001095752)	barB	Siderophore efflux system of the ABC superfamily [Acinetobactin]	Nutritional/Metabolic factor
VFG037274(gb|WP_001281538)	barA	Siderophore efflux system of the ABC superfamily [Acinetobactin]	Nutritional/Metabolic factor
VFG037288(gb|WP_000603876)	basG	Acinetobactin biosynthesis protein basf [Acinetobactin]	Nutritional/Metabolic factor
VFG037302(gb|WP_001018264)	basF	Aryl carrier protein BasF [Acinetobactin]	Nutritional/Metabolic factor
VFG037316(gb|WP_000744381)	entE	Non-ribosomal peptide synthetase adenylate-forming enzyme of acinetobactin synthesis [Acinetobactin]	Nutritional/Metabolic factor
VFG037330(gb|WP_001177743)	basD	Acinetobactin biosynthesis protein basd [Acinetobactin]	Nutritional/Metabolic factor
VFG037344(gb|WP_000717759)	basC	Acinetobactin biosynthesis protein basc [Acinetobactin]	Nutritional/Metabolic factor
VFG037358(gb|WP_001073030)	bauA	TonB-dependent siderophore receptor baua [Acinetobactin]	Nutritional/Metabolic factor
VFG037372(gb|WP_001104139)	bauB	Ferric siderophore ABC transporter, periplasmic siderophore-binding protein [Acinetobactin](	Nutritional/Metabolic factor
VFG037386(gb|WP_000582115)	bauE	Ferric siderophore ABC transporter, ATP-binding protein baue [Acinetobactin]	Nutritional/Metabolic factor
VFG037400(gb|WP_001223274)	bauC	Ferric siderophore ABC transporter, permease protein bauc [Acinetobactin]	Nutritional/Metabolic factor
VFG037414(gb|WP_012391956)	bauD	Ferric siderophore ABC transporter, permease protein baud [Acinetobactin]	Nutritional/Metabolic factor
VFG037428(gb|WP_000939834)	basB	Non-ribosomal peptide synthetase with condensation and peptidyl carrier protein domains [Acinetobactin]	Nutritional/Metabolic factor
VFG037442(gb|WP_000910253)	basA	Acinetobactin biosynthesis protein [Acinetobactin]	Nutritional/Metabolic factor
VFG037456(gb|WP_000160878)	bauF	Siderophore-interacting protein [Acinetobactin]	Nutritional/Metabolic factor
VFG037470(gb|WP_001105554)	ACICU_RS04565	Lysr family transcriptional regulator [Hem O cluster]	Nutritional/Metabolic factor
VFG037490(gb|WP_001240499)	ACICU_RS04575	FecR domain-containing protein [Hem O cluster]	Nutritional/Metabolic factor
VFG037497(gb|WP_085947929)	ACICU_RS04580	TonB-dependent receptor [Hem O cluster]	Nutritional/Metabolic factor
VFG037505(gb|WP_002002089)	ACICU_RS04585	Transferrin-binding protein-like solute binding protein [Hem O cluster]	Nutritional/Metabolic factor
VFG037513(gb|WP_085947930)	ACICU_RS04590	Porin family protein [Hem O cluster]	Nutritional/Metabolic factor
VFG037521(gb|WP_000837764)	ACICU_RS04595	TonB family protein [Hem O cluster]	Nutritional/Metabolic factor
VFG037599(gb|WP_001988023)	csuC	Csu pilus chaperone protein CsuC [Csu fimbriae]	Biofilm
VFG037612(gb|WP_000603301)	csuD	Csu pilus usher protein CsuD [Csu fimbriae]	Biofilm
VFG037625(gb|WP_001022730)	csuE	Csu pilus tip adhesin CsuE [Csu fimbriae]	Biofilm
VFG037664(gb|WP_001061322)	pgaB	Poly-beta-1,6-N-acetyl-D-glucosamine N-deacetylase [PNAG]	Biofilm
VFG037678(gb|WP_000866237)	pgaC	Poly-beta-1,6 N-acetyl-D-glucosamine synthase [PNAG]	Biofilm
VFG037705(gb|WP_000010636)	adeF	Membrane-fusion protein [AdeFGH efflux pump]	Biofilm
VFG037735(gb|WP_000633124)	adeH	Outer membrane protein [Ade FGH efflux pump ,	Biofilm
VFG037807(gb|WP_000013434)	lpxB	Lipid-A-disaccharide synthase [LPS]	Immune modulation
VFG037822(gb|WP_000240700)	lpxC	UDP-3-O-acyl-N-acetylglucosamine deacetylase [LPS	Immune modulation
VFG037852(gb|WP_001075965)	ACICU_RS00395	Polysaccharide biosynthesis tyrosine autokinase [Capsule]	Immune modulation
VFG037882(gb|WP_000872593)	ACICU_RS00405	Polysaccharide biosynthesis/export family protein [Capsule]	Immune modulation
VFG037897(gb|WP_001165095)	tviB	Vi polysaccharide biosynthesis UDP-N-acetylglucosamine C-6 dehydrogenase TviB [Capsule]	Immune modulation
VFG037912(gb|WP_000939040)	pseB	UDP-N-acetylglucosamine 4,6-dehydratase (inverting)[Capsule]	Immune modulation
VFG037927(gb|WP_000470518)	pseC	UDP-4-amino-4, 6-dideoxy-N-acetyl-beta-L-altrosamine transaminase [Capsule]	Immune modulation
VFG037957(gb|WP_000666850)	pseG	UDP-2,4-diacetamido-2,4, 6-trideoxy-beta-L-altropyranose hydrolase [Capsule]	Immune modulation
VFG037987(gb|WP_000037963)	pseI	Pseudaminic acid synthase [Capsule]	Immune modulation
VFG038002(gb|WP_000172211)	ACICU_RS00445	Hypothetical protein [Capsule]	Immune modulation
VFG038017(gb|WP_001010045)	ACICU_RS00450	Capsular polysaccharide synthesis protein [Capsule]	Immune modulation
VFG038062(gb|WP_000515955)	ACICU_RS00465	Hypothetical protein [Capsule]	Immune modulation
VFG038105(gb|WP_000591436)	galU	UTP--glucose-1-phosphate uridylyltransferase galu [Capsule]-	Immune modulation
VFG038119(gb|WP_000686134)	ACICU_RS00485	Nucleotide sugar dehydrogenase [Capsule]	Immune modulation
VFG038132(gb|WP_000045495)	pgi	Glucose-6-phosphate isomerase [Capsule]	Immune modulation
VFG038144(gb|WP_001062908)	galE	UDP-glucose 4-epimerase gale [Capsule]	Immune modulation
VFG038155(gb|WP_000209960)	ACICU_RS00500	Phosphomannomutase/phosphoglucomutase [Capsule]	Immune modulation
VFG038176(gb|WP_000777882)	ompA	Outer membrane protein Ompa [OmpA]	Immune modulation
VFG050372(gb|WP_001017033.1)	pilQ	Type IV pilus secretin PilQ [TFP]	Adherence
VFG050386(gb|WP_000355489.1)	pilT	Type IV pilus twitching motility protein PilT [TFP]	Adherence
VFG050457(gb|WP_001274990.1)	pilB	Type IV-A pilus assembly atpase PilB [TFP]	Adherence
VFG050470(gb|WP_000279216.1)	pilC	Type II secretion system F family protein [TFP]	Adherence
VFG050484(gb|WP_001152285.1)	gspO/pilD	A24 family peptidase [TFP]	Adherence
VFG050540(gb|WP_000079192.1)	pilW	PilW family protein [TFP	Adherence
VFG050567(gb|WP_000768964.1)	pilY1	VWA domain-containing protein [TFP]	Adherence
VFG050670(gb|WP_000505931.1)	pilJ	Methyl-accepting cHem O taxis protein [TFP]	Adherence
VFG050685(gb|WP_001160337.1)	pilS	PAS domain-containing sensor histidine kinase [TFP]	Adherence
VFG050727(gb|WP_000870033.1)	gspC	General secretion pathway protein C [T2SS]	Effector delivery system
VFG050742(gb|WP_001196426.1)	gspD	General secretion pathway protein D [T2SS]	Effector delivery system
VFG050772(gb|WP_001020922.1)	gspE2	Putative secretion pathway ATPase [T2SS]	Effector delivery system
VFG050860(gb|WP_000301479.1)	gspK	General secretion pathway protein K [T2SS]	Effector delivery system
VFG050901(gb|WP_005119983.1)	cpaA	Metalloendopeptidase cpaa [CpaA]	Effector delivery system
VFG050910(gb|WP_001178477.1)	vgrG/tssI	Type VI secretion system tip protein VgrG [T6SS]	Effector delivery system
VFG050911(gb|WP_000934999.1)	vgrG/tssI	Type VI secretion system tip protein VgrG [T6SS]	Effector delivery system
VFG050912(gb|WP_000898330.1)	vgrG/tssI	Type VI secretion system tip protein VgrG [T6SS]	Effector delivery system
VFG050946(gb|WP_000083625.1)	tssL	Type IVB secretion system protein TssIL [T6SS]	Effector delivery system
VFG050959(gb|WP_000556915.1)	tssM	Type VI secretion system membrane subunit tssm [T6SS]	Effector delivery system
VFG050972(gb|WP_000972583.1)	tagX	Type VI-associated gene X, peptidoglycan hydrolase [T6SS]	Effector delivery system
VFG051000(gb|WP_000568832.1)	tssF	Type VI secretion system baseplate subunit TssF [T6SS]	Effector delivery system
VFG051014(gb|WP_001190395.1)	tssG	Type VI secretion system baseplate subunit TssG [T6SS]	Effector delivery system
VFG051028(gb|WP_000471445.1)	tssK	Type VI secretion system baseplate subunit TssK[T6SS]	Effector delivery system
VFG051070(gb|WP_001066523.1)	tssC	Type VI secretion system contractile sheath large subunit [T6SS]	Effector delivery system
VFG051084(gb|WP_000020713.1)	tssA	Type VI secretion system protein TssA [T6SS]	Effector delivery system
VFG051098(gb|WP_002001416.1)	clpV/tssH	Type VI secretion system atpase TssH [T6SS]	Effector delivery system
VFG051109(gb|YP_001083177.1)	tse1	T6SS Tse1, predicted lipase [T6SS secreted Effectors]	Effector delivery system
VFG051110(gb|YP_001083606.1)	tse2	T6SS Tse2, predicted nuclease [T6SS secreted Effectors]	Effector delivery system
VFG051111(gb|YP_001084320.1)	tse3	T6SS Effector Tse3, unknown function [T6SS secreted Effectors]	Effector delivery system
VFG037028(gb|WP_002218620)	katA	Catalase [katA]	Stress survival
VFG001223(gb|NP_249086)	pilT	Twitching motility protein PilT [Type IV pili]	Adherence

S_Table_3B. Virulence genes of *A. pittii*, belonging to the accessoty genome, found in the VFDB database.
