# Supplementary material for: Acinetobacter pittii: the emergence of a hospital-acquired pathogen analyzed from the genomic perspective
Source: Front Microbiol. 2024 Jun 26;15:1412775. doi: 10.3389/fmicb.2024.1412775 (PMC11233732; doi:10.3389/fmicb.2024.1412775)
Supplement: Supplementary file 10 [file Data_Sheet_10.pdf]

Rep (Protein_ID)	Rep_protein	Plasmid	Accession_number	Plasmid lineage	Host_Range
WP_002046604.1	RepM_Acin	p11	NZ_CP021429.1	ORPHAN	*A. baumannii, A. berenziniae, A. cumulans, A. guerrae, A. haemolyticus, A. junii, A. lactucae, A. modestus, A. nosocomilais, A. pittii, A. proteolyticus, A. seifertii, A. ursinglii, A. venetianus, A. wuhouensis, Flavobacterium johnsoniae, Salmonella enterica*
		pOXA58_100020	NZ_CP027253.1	ORPHAN	
		pOXA58_005046	NZ_CP028573.2	ORPHAN	
		pAB17H194-2	NZ_CP040913.1	ORPHAN	
		pWP2-W18-ESBL-11_1	NZ_AP021937.1	ORPHAN	
		pTCM-1	NZ_CP095408.1	PLP_5	
		pAP8900-1	NZ_CP123766.1	PLP_5	
		unnamed	NZ_CP029611.1	PLP_5	
		pOXA58_100004	NZ_CP027249.2	PLP_12	
		pApW20-2	NZ_CP027660.1	PLP_14	
		pAbW39-2	NZ_CP029007.1	PLP_14	
WP_000064928.1	Rep_3-DUF5710	pMX2	NZ_CM001803.1	PLP_2	*A. baumannii, A. junii, A. lactucae, A. nosocomialis, A. oleivorans, A. pittii, A. soli, A. tandoii,*
		p1_005069	NZ_CP026087.2	PLP_2	
		p1_100004	NZ_CP027247.2	PLP_2	
		p1_100020	NZ_CP027251.3	PLP_2	
		pC54_002	NZ_CP042366.1	PLP_2	
		pAP43-2	NZ_CP043054.1	PLP_2	
		unnamed2	NZ_CP069506.1	PLP_2	
		unnamed3	NZ_CP069540.1	PLP_2	
		unnamed1	NZ_CP077304.1	PLP_2	
		pCEP14_01	NZ_CP084922.1	PLP_2	
WP_005804946.1	RepM_Acin	pOXA58-AP_882	NZ_CP014479.1	ORPHAN	*A. baumannii, A. bereziniae, A. colistiniresistens, A. gerneri, A. haemolyticus, A. lactucae, A. nosocomialis, A. piscicola, A. pittii, A. proteolyticus, A. rongchengensis, A. schindleri, A. seifertii, A. soli, A. ursingli*
		pA1254_4	NZ_CP049810.1	ORPHAN	
		p2_005046	NZ_CP028570.1	ORPHAN	
		pCEP14_02	NZ_CP084923.1	ORPHAN	
		p2_100020	NZ_CP027252.3	PLP_3	
		unnamed1	NZ_CP069497.1	PLP_3	
		unnamed3	NZ_CP069507.1	PLP_3	
		unnamed1	NZ_CP069538.1	PLP_3	
		pML4-2	NZ_CP118935.1	PLP_3	
WP_005065049.1	RepM_Acin	pAP2044-4	NZ_CP027662.1	ORPHAN	*A. baumannii, A. bereziniae, A. lowffii, A. nosocomialis, A. pittii, A. seifertii, A. variabilis*
		pOCUAc17-3	NZ_AP024801.1	PLP_6	
		unnamed2	NZ_CP077240.1	PLP_6	
		unnamed1	NZ_CP069505.1	PLP_6	
		pAbW39-2	NZ_CP029007.1	PLP_14	
		unnamed2	NZ_CP069498.1	ORPHAN	
WP_000818856.1	RepM_Acin	p1_005046	NZ_CP028569.1	PLP_7	*A. baumannii, A. geminorum, A. nosocomialis, A. pittii*
		unnamed1	NZ_CP077239.1	PLP_7	
		pML4-1	NZ_CP118934.1	PLP_7	
WP_000845851.1	RepM_Acin	pAP43-3	NZ_CP043055.1	PLP_9	*A. baumannii, A. nosocomialis, A. oleivorans, A. pittii, A.seifferti, A. stercoris, Klebsiella pneumoniae, Methylococcus capsulatus, Sphingobium*
		p2_005069	NZ_CP026088.1	PLP_9	
		pOXA58_005069	NZ_CP026086.2	PLP_12	
WP_004843977.1	RepM_Acin	pCEP14_03	NZ_CP084924.1	ORPHAN	
		pAP8900-2	NZ_CP123767.1	PLP_11	*A. baumannii, A. junii, A. pittii, A. seifertii*
		pAP2044-2	NZ_CP087718.1	PLP_11	
WP_005133531.1	RepM_Acin	pA1254_3	NZ_CP049809.1	ORPHAN	
		pTCM-2	NZ_CP095409.1	PLP_8	*A. baumannii, A. pittii, A. seifertii, Klebsiella pneumonie*
		pAP8900-4	NZ_CP123769.1	PLP_8	
		unnamed4	NZ_CP069508.1	PLP_10	
		unnamed2	NZ_CP077305.1	PLP_10	
WP_078220828.1	II	unnamed1	NZ_CP017939.1	ORPHAN	*A. pittii*
WP_057082420.1	Replicase-PriCT-HTH-23	p3_005046	NZ_CP028571.1	ORPHAN	*A. baumannii, A. nosocomialis, A. pittii, A. ursingli*
WP_032071712.1	Rep_1	p4_005046	NZ_CP028572.2	ORPHAN	*A. baumannii, A. pittii, Escherichia coli, Klebsiella pneumonie*
WP_001031297.1	Rep_3	pC54_004	NZ_CP042368.1	ORPHAN	*A. baumannii, A. colistiniresistens, A. defluvi, A. johnsonii, A. lactucae, A. lani, A. lwoffii, A. nosocomialis, A. pittii, A. pseudolwoffii, A. schindleri, A. ursinglii, wuhouensis*
WP_150378260.1	Rep_3-DUF5710	pAB17H194-1	NZ_CP040912.1	ORPHAN	*A. baumannii, A. cumulans, A. indicus, A. pittii, A. townwri*
WP_167564503.1	Rep_3	pA1254_2	NZ_CP049808.1	ORPHAN	*A. pittii*
WP_001180320.1	Rep1	pWP2-W18-ESBL-11_3	NZ_AP021939.1	ORPHAN	*A. baumannii, A. geminorum, A. nosocomialis, A. pittii, Burkholderia cepacia, Staphyloccocus aurus*
WP_004795963.1	Rep_3-DUF5710	pOCUAc17-1	NZ_AP024799.1	ORPHAN	*A. nosocomialis, A. pittii*
WP_004728629.1	Rep_3	pTCM-3	NZ_CP095410.1	ORPHAN	*A. baumannii, A. bereziniae, A. cumulans, A. johnsonii, A. lwoffii, A. nosocomialis, A. piscicola, A. pittii, A. radioresistans, A. soli, A. tjernbergiae, A. wuhouensis, Campilobacter jejuni, Escherichia coli, Streotococcus pneumonie*
WP_069120316.1	CyRepA1	pAP2044-2	NZ_CP087718.1	PLP_11	*A. baumannii, A. pittii, A. seifertii*
WP_004698334.1	RepM_Acin	p2_100004	NZ_CP027248.1	ORPHAN	*A. baumannii, A. nosocomialis, A. pittii, A. seifertii, A. ursinglii*
WP_004763412.1	RepM_Acin	pOCUAc17-2	NZ_AP024800.1	ORPHAN	*A. baumannii, A. johnsonii, A. oleivorans, A. pittii, Lactococcus*
WP_012780181.1	RepM_Acin	pIEC338SCOX	NZ_CP015146.1	ORPHAN	*A. baumannii, A. baylyi, A. calcoaceticus, A. bereziniae, A. haemolyticus, A. nosocomialis, A. pittii, A. wuhouensis*
WP_016803254.1	RepM_Acin	unnamed2	NZ_CP069539.1	ORPHAN	*A. pittii, A. ursingli*
WP_063099738.1	RepM_Acin	pIEC338SC2	NZ_CP015147.1	ORPHAN	*A. pittii*
WP_063099746.1	RepM_Acin	pIEC338SC3	NZ_CP015148.1	ORPHAN	*A. pittii*
WP_114225253.1	RepM_Acin	pC54_003	NZ_CP042367.1	ORPHAN	*A. baumannii, A. pittii*
WP_167564445.1	RepM_Acin	pA1254_1	NZ_CP049807.1	ORPHAN	*A. pittii*
WP_171258818.1	RepM_Acin	p5_UKK-0548	NZ_MDIM02000005.1	ORPHAN	*A. oleivorans, A. pittii*
WP_187406175.1	RepM_Acin	p1	NZ_CP107290.1	ORPHAN	*A. baumannii, A. pittii*
WP_252618463.1	RepM_Acin	pApW20-3	NZ_CP027661.1	ORPHAN	*A. pittii*
WP_004843977.1	RepM_Acin	pCEP14_03	NZ_CP084924.1	ORPHAN	*A. baumannii, A. junii, A. pittii, A. seifertii*
WP_016803254.1	RepM_Acin	unnamed2	NZ_CP069539.1	ORPHAN	*A. pittii, A. ursingli*
WP_114225253.1	RepM_Acin	pC54_003	NZ_CP042367.1	ORPHAN	*A. baumannii, A. pittii*
WP_167564445.1	RepM_Acin	pA1254_1	NZ_CP049807.1	ORPHAN	*A. pittii*
WP_216972120.1	RepM_Acin	unnamed3	NZ_CP077241.1	ORPHAN	*A. pittii*
WP_004896921.1	RepM_Acin	pApiHUM1a	NZ_CP139265.1	ORPHAN	*A. baumannii, A. indicus, A. kyonggiensis, A. lwoffii, A. nosocomialis, A. oleivorans, A. pittii, A. schindleri, A. ursingli*
WP_005244554.1	RepM_Acin	pApiA45Pa	NZ_CP139359	ORPHAN	*A. pittii, A. geminorum, A. piscicola, A. soli, A. venetianus*
WP_026441267.1	RepM_Acin	pApiMCR16048a	NZ_CP139257.1	ORPHAN	*A. baumannii, A. johnsonii, A. junii, A. pittii, A. tandoii*
WP_032066855.1	RepM_Acin	pApiHUM1b	NZ_CP139263.1	ORPHAN	*A. baumannii, A. bereziniae, A. guillouiae, A. johnsonii, A. nosocomialis, A. pittii, A. ursinglii, A. toyonenesis, Finigoldia magna*
		pApiAE13b	NZ_CP139284.1	ORPHAN	
WP_032859646.1	RepM_Acin	pApiAE13a	NZ_CP139285.1	ORPHAN	*A. baumannii, A. bereziniae, A. colistiniresistens, A. haemolyticus, A. higginsii, A. pittii, A. ursinglii,*
WP_034700330.1	Rep_3_DUF5710	pApiHCG62c	NZ_CP139272.1	ORPHAN	*A. gerneri, A. johnsonii, A. pittii, A. A. venetianus*
WP_048766123.1	RepM_Acin	pApiA45Pb	NZ_CP139358	ORPHAN	*A. baumannii, A. bereziniae, A. lactucae, A. pittii, A. ursinglii*
WP_057082420.1	Replicase-PriCT-HTH-23	p3_005046	NZ_CP028571.1	ORPHAN	*A. nosocomialis, A. pittii, A. ursinglii*
WP_063099738.1	RepM_Acin	pIEC338SC2	NZ_CP015147.1	ORPHAN	*A. pittii*
WP_104039705.1	RepM_Acin	pApiHUM14b	NZ_CP139268.1	ORPHAN	*A. baumannii, A. pittii*
WP_114225253.1	RepM_Acin	pC54_003	NZ_CP042367.1	ORPHAN	*A. baumannii, A. pittii*
WP_199953184.1	RepM_Acin	pAP8900-3	NZ_CP123768.1	ORPHAN	*A. pittii*
WP_226789443.1	CyRepA1	pCEP14_03	NZ_CP084924.1	ORPHAN	*A. pittii*
WP_320561151.1	Replicase-PriCT	pApiAN37b	NZ_CP139280.1	ORPHAN	*A. pittii*
WP_320562525.1	RepM_Acin	pApiMCR53b	NZ_CP139253.1	ORPHAN	*A. pittii*

S_Table_4. The first column shows the GenBank accession number of the Rep protein, and its classification based on its protein domains (second column). The third column shows the plasmid names containing such Rep proteins and their GenBank accession numbers. The penultimate column indicates whether the plasmid belongs to a lineage (PLP) or not (ORPHAN). Next to the last column shows the species in which we found plasmids with proteins with identical Rep proteins, i.e., with the same accession number of the protein.
